# Fabrication and optimization of magnetic amino-functionalized polyacrylonitrile nanocomposites for enhanced copper removal from aqueous media

**DOI:** 10.1038/s41598-025-33675-3

**Published:** 2026-01-18

**Authors:** Marwa A. Moharram, Mohamed A. Salem, Murat Yılmaz, Mohamed A. Hassaan, Mohamed A. El-Nemr, Ahmed El Nemr

**Affiliations:** 1 Chemistry Department, Faculty of Science, Tanta University, Tanta, 31527 Egypt; 2 Department of Chemistry and Chemical Processing Technologies, Bahçe Vocational School, Osmaniye Korkut Ata University, Osmaniye, 80000 Turkey; 3 National Institute of Oceanography and Fisheries (NIOF), Kayet Bey, Elanfoushy, Alexandria Egypt; 4 Department of Chemical Engineering, Faculty of Engineering, Minia University, Minia, 61519 Egypt; 5 The Higher Canal Institute of Engineering and Technology, Al Salam 1 - Abu Bakr Al Siddiq Street, Suez, Egypt

**Keywords:** Adsorption, Magnetite adsorbent, Amino polymer, Nanocomposite, Copper removal, Water treatment, Chemistry, Environmental sciences, Materials science, Nanoscience and technology

## Abstract

A magnetic amino polyacrylonitrile nanocomposite (MAPA) was prepared and applied as an adsorbent to eliminate Cu^2+^ ions from aqueous media through batch experiments. Its physicochemical properties were examined using most known characterization methods. The optimal removal efficiency was obtained at pH 5.5. Adsorption studies were performed under different experimental conditions, considering initial copper concentration, solution pH, and temperature. The maximum removal efficiency attained was 78.29%, while the corresponding maximum adsorption capacity ($\:{q}_{m}$) was calculated to be 5.65 mg/g. The equilibrium adsorption data were analyzed using different isotherm models, among which the Langmuir (LIM) exhibited the best correlation with the experimental results. Kinetic behavior was also assessed through various known models. Among these models, the pseudo-second-order (PSO) demonstrated the strongest correlation (*R*² = 1.0), indicating that it most precisely represents the adsorption behavior. In summary, the fabricated MAPA demonstrated strong potential for efficiently eliminating Cu^2+^ ions from aqueous solutions. A maximum removal percentage of 51.51 mg/L of Cu^2+^ ions and 5.12 g of MAPA could be attained by using a Response Surface Methodology (RSM) optimization of the adsorption parameters. The optimized BPNN of ANN model stopped after 4 epochs with the best validation of 5.474, with an overall *R*^2^ of 0.996.

## Introduction

Water pollution is a critical worldwide concern due to its adverse health impacts^1^. Heavy metals are among the major contributors to this problem, originating from diverse anthropogenic sources^2,3^. Such sources include industrial operations (e.g., metallurgy and manufacturing), agricultural runoff, improper waste management, and natural geochemical activities. Copper ions (Cu^2+^) are particularly prevalent among these metals, commonly detected in street dust and industrial wastewater discharges^4^. Elevated concentrations of Cu^2+^ ions are of particular concern due to their harmful impacts on the nervous system. Owing to its extensive use in diverse fields - such as electrical wiring, heat transfer systems, catalysts, and pulp processing - copper is frequently released into the environment. Various approaches have been employed to address heavy metal pollution, including precipitation, ion exchange, and reverse osmosis^5–8^. Among these, adsorption is acknowledged as a straightforward yet highly efficient method, especially for removing of contaminants at trace concentrations^9–11^.

Consequently, considerable research has been directed toward developing advanced adsorbent materials. Among them, polymer-based nanocomposites show great potential because their high surface area, suitable pore structure, and strong mechanical stability enhance adsorption efficiency. In addition, they are relatively inexpensive, easy to handle, and can be regenerated under mild conditions^12,13^.

Polyacrylonitrile (PAN), a potential synthetic polymer, is primarily studied to develop novel nanocomposites across diverse sectors, including biomedicine, textiles, automotive industries, and electronics. PAN and its composites have garnered significant attention due to their distinctive characteristics, including rigidity, low weight, and high strength^14–16^.

A multifunctional dysprosium oxide–biochar–montmorillonite composite with exceptional adsorption and recyclability toward cationic contaminants was synthesized by Yang et al. in 2025^17^. Their research emphasizes how crucial it is for inorganic oxides and bio-based supports to act in concert to achieve high adsorption efficiency and regeneration capacity. Developing magnetic polymer-based adsorbents with improved recovery and reuse performance is strongly justified by using this information. Xue et al. (2025) described a bio-based benzoxazine–phthalonitrile polymer demonstrating better stability and structural integrity, emphasizing the relevance of polymer design in developing durable and efficient materials for environmental applications^18^. This is consistent with the current study’s strategy of adding amino groups to polyacrylonitrile to enhance surface reactivity and thermal stability for copper ion adsorption. A 3D PVA/GO/ZIF-67 cryogel with remarkable porosity and adsorption capacity for Cd^2+^ and Pb^2+^ ions was created by Motaghi et al. (2022), demonstrating how polymer–oxide hybrids can improve adsorption through synergistic porosity and surface functionality^19^. The study’s approach to combine polymer matrices with magnetic nanoparticles for better heavy metal removal is supported by the incorporation of comparable composite principles. Surface functionalization dramatically increases metal binding affinity, according to Esfandian et al. (2013), who investigated copper adsorption using modified brown algae and compared batch versus column performance^20^. Their findings emphasize the relevance of incorporating amino functionalities in adsorbent to boost Cu^2+^ ion adsorption efficiency.

Verma et al. (2022) reported the adsorption of metal ions and MO dye were performed with the newly synthesized magnetite-doped CS-EDTA composite^21^. The adsorption of Pb(II), Cd(II), and Cu(II) onto the developed composite CS-EDTA was investigated by performing batch experiments^22^. Graphene oxide was successfully functionalized on the multifunctional *β*-cyclodextrin chitosan polymer through EDTA crosslinking for the adsorption of toxic pollutant^23,24^. A trifunctional *β*-cyclodextrin-ethylenediaminetetraacetic acid-chitosan polymer was synthesized using an easy and simple chemical route by the reaction of activated *β*- cyclodextrin with chitosan through EDTA as a cross-linker (amidation reaction) for the removal of inorganic and organic pollutants from aqueous solution under different parameters^25^.

A significant limitation of conventional adsorbents is their challenging recovery from treated water, often necessitating rapid centrifugation or fine filtration techniques. Magnetic adsorbents overcome this drawback, as they can be conveniently retrieved from solution by applying an external magnetic field^26,27^.

The present study is devoted to designing a new adsorbent from 3-aminopropyl trimethoxysilane and testing the formatted magnetic amino polyacrylonitrile nanocomposite to remove Cu^2+^ ions from contaminated water. Attention is given to evaluating the influence of operational parameters, including adsorbent dosage, contact duration, and solution pH, on the adsorption performance. Furthermore, adsorption mechanisms and kinetics are examined using different theoretical models to better understand the process. RSM and ANN models were used to optimize the adsorption of Cu^2+^ ions from wastewater. The overarching objective is to introduce a sustainable and innovative approach for industrial wastewater treatment, thereby reducing the adverse impacts of copper pollution.

## Materials and methods

### Materials and equipment

Ethanol (C_2_H_5_OH, 99.99%) and ammonia solution (25%) were obtained from the International Company for Sup. & Med. Industries, Egypt. Acrylonitrile stabilized (99%) was procured from Loba Chemie. Ferrous sulfate heptahydrate (98.5%) was purchased from Alpha Chemical, India, while ferric chloride (98.5%) and copper sulfate (CuSO_4_, 99%) were supplied by Fisher Scientific, UK. In addition, 3-aminopropyl trimethoxysilane (95%) was sourced from Acros Organics, *N*,*N*-dimethylformamide (DMF, 99.8%) from ADVENT Chembion Pvt. Ltd., India, and ammonium persulfate (98%) from Oxford Lab Chem, India.

The pollutant concentrations were quantified using a UV–visible spectrophotometer (Analytic Jena, SPEKOL 1300) fitted with 1.0 cm optical path length glass cuvettes. The experimental work was carried out with a SANYO microwave oven (EM-D975W, 1400 W maximum input), a JENCO pH meter (6173), a VELP magnetic stirrer with heating function (Code F20500010), and a JS orbital shaker (JSOS-500). The surface functional groups of the adsorbent were characterized using Fourier Transform Infrared (FTIR) spectroscopy using a platinum attenuated total reflection (ATR) unit (VERTEX 70, Model V-100) over the spectral range of 400–4000 cm^–1^. Surface morphology and elemental distribution were further examined through Scanning Electron Microscopy (SEM, LEO 1450 VP) combined with Energy Dispersive X-ray Spectroscopy (EDAX).

### Preparation of magnetic amino polyacrylonitrile nanocomposite (MAPA)

#### Preparation of polymer

A solution was prepared by dissolving 9 mL of acrylonitrile in *N*,*N*-dimethylformamide to a final volume of 25 mL. Ammonium persulfate served as the polymerization initiator. Within the initial few minutes of the reaction, 5 mL of 3-aminopropyl trimethoxysilane was gradually introduced. The polymerization was then maintained at 70 °C under continuous stirring for three hours. The resultant precipitate was collected, thoroughly washed with distilled water, and dried at 50 °C (95% yield).

#### Preparation of magnetic nanopolymer

Magnetite was incorporated into the polymer through the co-precipitation method, a widely used approach for synthesizing magnetic nanoparticles^28^. In this procedure, the preformed polymer was adjusted to pH 11 and combined in a conical flask with two separate solutions: FeSO_4_·7H_2_O (6.75 g in 100 mL distilled water) and FeCl_3_ (8.0 g in 100 mL distilled water). The reaction mixture underwent sonication for 1.5 h, followed by the separation of the magnetic polymer, repeatedly washed with distilled water, and subsequently dried at 50 °C (92% yield). The final product was then stored in a sealed container until further use.

### Adsorption study

The removal performance of copper ions from aqueous solutions was assessed through batch adsorption experiments. All investigations were conducted at ambient temperature (25 °C) with constant agitation on a shaking apparatus. Solution pH was regulated by adding 0.1 M HCl or NaOH. A predetermined amount of the synthesized magnetic amino polyacrylonitrile nanocomposite (MAPA) adsorbent was placed in flasks, followed by the addition of solutions with different initial Cu^2+^ ion concentrations. To examine the effect of operating conditions on adsorption performance, adsorbent dosage (2.0, 2.5, 3.0, 4.0, 5.0, and 6.0 g/L), contact time (0–30 min), solution pH (1–5), and initial Cu^2+^ ion concentrations (50, 75, 100, and 150 mg/L) were systematically examined.

The concentrations of Cu^2+^ ion at both the initial and equilibrium stages were determined using a UV–Vis spectrophotometer at the maximum absorption wavelength (*λ*_max_ = 460 nm)^29,30^. Samples were subjected to shaking at 200 rpm, and at specified time intervals, 1.0 mL aliquots of the supernatant were collected for analysis. All adsorption experiments were conducted in triplicate, with the reported data representing the mean values. The percentage removal of Cu^2+^ ion (%*R*) was calculated according to Eq. (1)^26^.

1 $$\:\%R=\frac{{C}_{i}-{C}_{e}}{{C}_{i}}\times\:100$$

where, *C*_*i*_ and *C*_*e*_ denote the Cu^2+^ ion concentrations (mg/L) at the initial and equilibrium adsorption states, respectively. The amount of Cu^2+^ adsorbed at a given time *t* (min) on the magnetic amino polyacrylonitrile nanocomposite (*Q*_t_) was calculated using Eq. (2):

2 $$\:{Q}_{t}=\frac{\left({C}_{i}-{C}_{t}\right)}{W}\times\:V$$

where, *C*_t_ (mg/L) signifies the concentration of Cu^2+^ ions at time *t*, *V*(L) indicates the volume of the original feed solution, and *W* (g) refers to the mass of the magnetic amino polyacrylonitrile nanocomposite (MAPA) employed as an adsorbent. The equilibrium adsorption capacity *Q*_e_ (mg/g) for the produced MAPA nanocomposite was ascertained utilizing Eq. (3):

3 $$\:{Q}_{e}=\frac{({C}_{0}-{C}_{e})\times\:V}{W}$$

Where, *C*_0_ (mg/L) and *C*_e_ (mg/L) denote the initial and the equilibrium concentrations of Cu^2+^ ions, respectively. *W* (g) signifies the weight of the adsorbent, whereas *V* (L) indicates the volume of the Cu^2+^ solution. The experiments were repeated three times and the standard deviation was ≤ 2.45.

### ANN modelling

The prediction of relationships between input and output data is achieved through biological human brain networks, a process known as ANN modelling. Feed-forward back-propagation neural networks (BPNN) are the most common type. They consist of three main components: an input layer (IL) (independent variable), hidden layers (HNs), and an output layer (OL) (dependent variable). MATLAB R2015b utilizes the Levenberg-Marquardt (LM) training algorithm to model Cu (II) removal by MAPA. The LM training algorithm uses training data (70%), validation data (15%), and testing data (15%). The optimal BPNN includes a hidden layer (HL) with 5 neurons. The inputs are the adsorbent dosage of MAPA (g/L), time (min), and the initial concentration of Cu^2+^ (mg/L), while the removal of Cu^2+^ is the output variable (Table 1)^31,32^.

**Table 1:** Data for the removal of Cu^2+^ ions using MAPA adsorbent.

Run	Factor 1	Factor 2	Factor 3	Response 1	ANN	RSM
	A: MAPA dose	B: Time	C: Cu dose	Cu ions removal	Prediction	Prediction
	g/L	min	mg/L	%	%	%
1	2	60.0	100	38.45	38.45	38.33
2	4	60.0	150	22.64	22.23	22.31
3	6	1.0	100	35.65	35.26	35.77
4	4	30.5	100	36.66	36.97	36.66
5	6	30.5	50	73.087	72.89	72.63
6	2	30.5	50	54.23	54.42	54.42
7	4	30.5	100	36.66	36.97	36.66
8	4	30.5	100	36.66	36.97	36.66
9	2	30.5	150	16.77	16.05	17.23
10	4	30.5	100	36.66	36.97	36.66
11	4	1.0	150	14.76	14.20	14.82
12	4	1.0	50	53.11	53.76	53.44
13	2	1.0	100	22.66	22.62	22.14
14	6	60.0	100	45.89	45.36	46.41
15	4	60.0	50	72.84	71.56	72.78
16	4	30.5	100	36.66	36.97	36.66
17	6	30.5	150	20.92	20.95	20.73

### RSM

Response surface methodology (RSM) was used to study the optimization of factors affecting Cu^2+^ ion removal in the presence of magnetic amino polyacrylonitrile nanocomposite (MAPA). The Box-Behnken design (BBD) was used, and Design-Expert version 13.0.5.0 was the software used. The starting Cu^2+^ ion concentration, adsorbent dose, and reaction duration were chosen. Table 2 lists the parameters under study along with their corresponding levels. Cu^2+^ ion removal percentage (%) was the answer under investigation. Seventeen tests were carried out using various combinations of factors.

**Table 2:** A range of experimental parameters considered in the optimization study.

Factor	Name	Units	Minimum	Maximum	Mean	Std. Dev.
A	Dose	g/L	2.00	6.00	4.00	1.41
B	Time	Min	1.00	60.00	30.50	20.86
C	Cu^2+^ Conc.	mg/L	50.00	150.00	100.00	35.36

## Results and discussion

### Characterization of magnetic amino poly-acrylonitrile nanocomposite (MAPA)

#### Scanning electron microscopy (SEM)

**Fig. 1:**
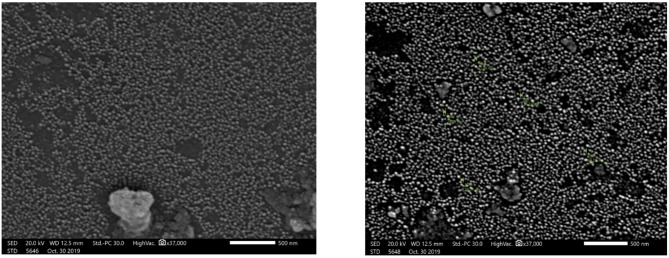
SEM images of magnetic amino polyacrylonitrile nanocomposite (MAPA) adsorbents.

Figure 1 illustrates the Scanning Electron Microscopy (SEM) images of the magnetic amino polyacrylonitrile nanocomposite. The micrographs reveal well-defined, spherical nanoparticles with an average size ranging from 21.49 to 28.27 nm.

#### Thermal gravimetric analysis (TGA) of MAPA

**Fig. 2:**
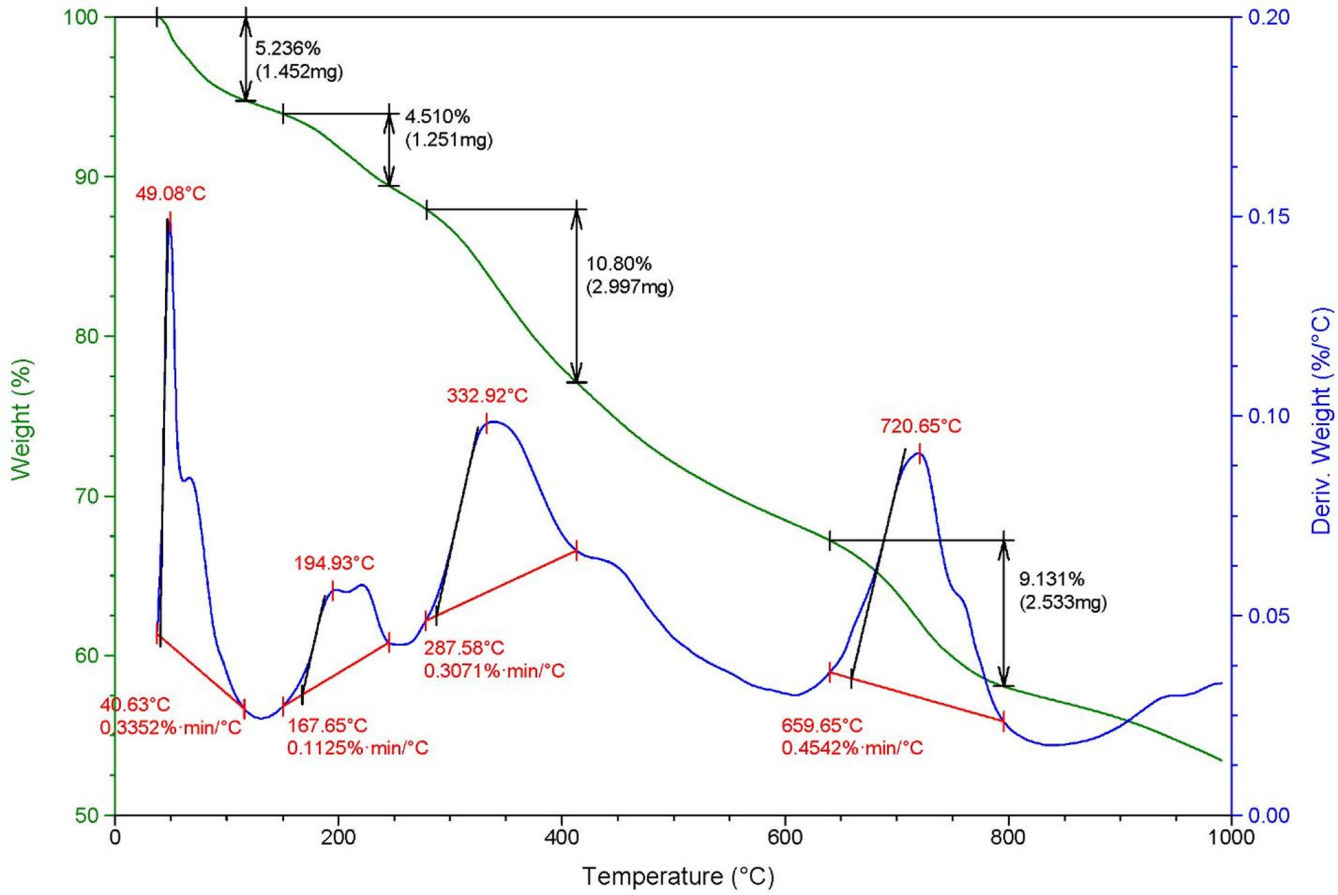
TGA and DTA graph of magnetic amino polyacrylonitrile nanocomposite (MAPA) adsorbents.

Thermogravimetric analysis (TGA) was conducted to assess the thermal stability of the magnetic amino polyacrylonitrile nanocomposite. This technique, which records the mass change of a sample upon heating, indicated multiple stages of decomposition. The initial weight loss of 5.24% occurred below 167 °C, corresponding to releasing physically and chemically bound water molecules. A subsequent decrease of 4.51% was detected between 195 and 250 °C, associated with depolymerization and degradation of polymer chains. The third stage, showing a 10.80% loss, was observed in the 300–400 °C range, while the fourth stage exhibited a 9.13% reduction between 660 and 800 °C. At 1000 °C, the overall mass loss reached 30%. These findings provide insight into the material’s decomposition profile and confirm its thermal stability across different temperature intervals (Fig. 2).

#### FTIR of magnetic amino poly-acrylonitrile nanocomposite (MAPA)

**Fig. 3:**
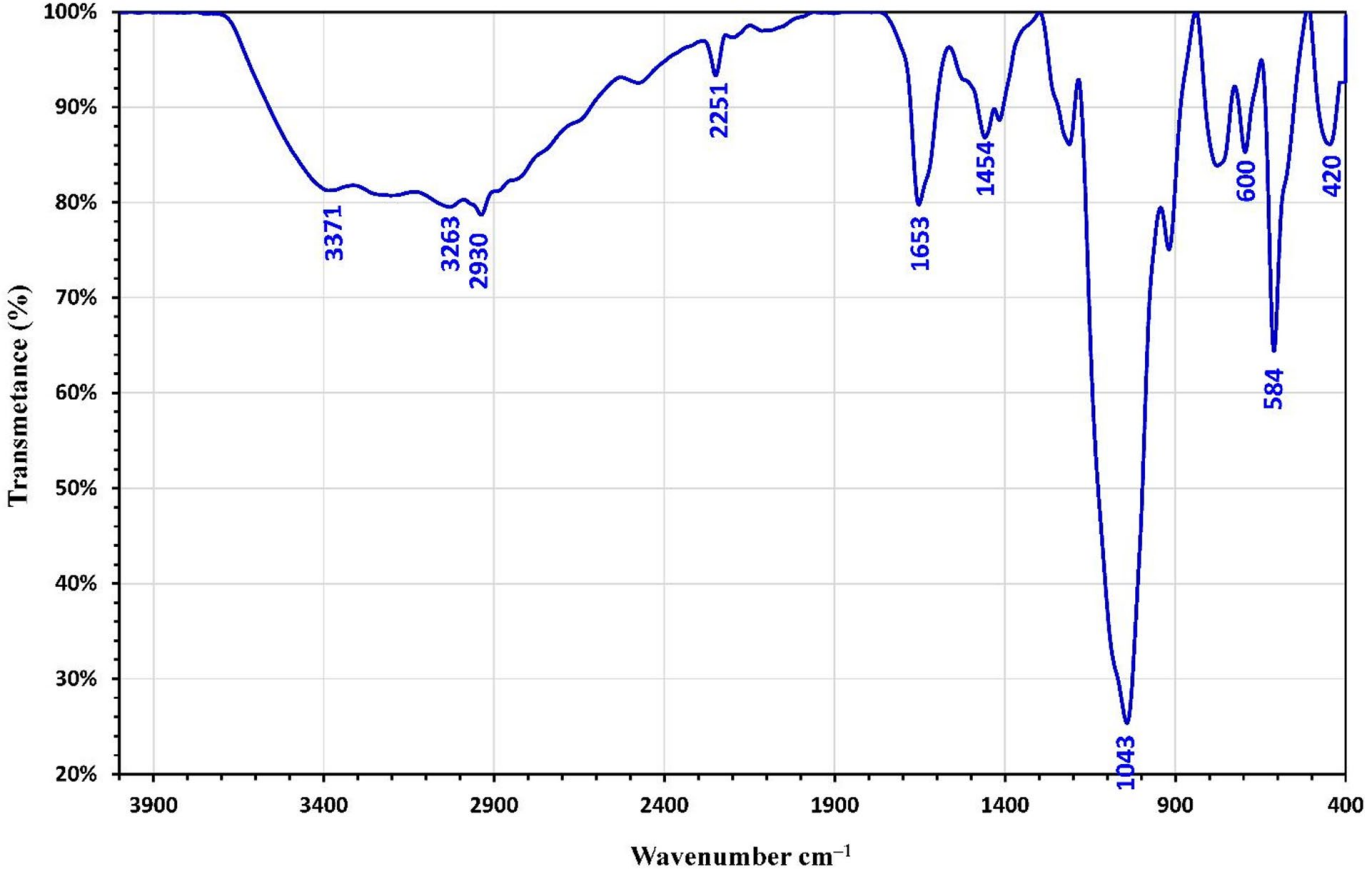
FTIR analysis of magnetic amino polyacrylonitrile (MAPA) nanocomposite adsorbent.

Fourier Transform Infrared (FTIR) spectroscopy was used to characterize the magnetic amino polyacrylonitrile nanocomposite, as it is a reliable method for identifying specific functional groups^33^. The FTIR spectrum of the nanocomposite is shown in Fig. 3. A broad absorption band around 3263 cm^–1^ is ascribed to the –OH stretching vibrations of hydroxyl groups on the surface of the magnetic nanoparticles. Peaks corresponding to Fe–O stretching in magnetite are evident within the 400–600 cm^–1^ range^34,35^, with prominent signals at 584 and 420 cm^–1^ confirming the presence of magnetite in the composite^36,37^. The C = C stretching vibration is indicated by a band at 1448 cm^–1^^38^, while the C–H stretching vibration of the CH_2_ groups in the polymer chain appears at 2930 cm^–1^. Bending vibrations of CH_2_ groups are detected in the fingerprint region at 1414 and 1454 cm^–1^. The N–H stretching and bending vibrations are observed at 3371 and 1653 cm^–1^, respectively^39^. The C–N stretching, representing the linkage of the amine group to the polymer backbone, appears at 1043 cm^–1^, and a band at 2251 cm^–1^ is assigned to C ≡ N stretching vibrations^33,40^.

#### BET surface area of magnetic amino poly-acrylonitrile (MAPA) nanocomposite

**Fig. 4:**
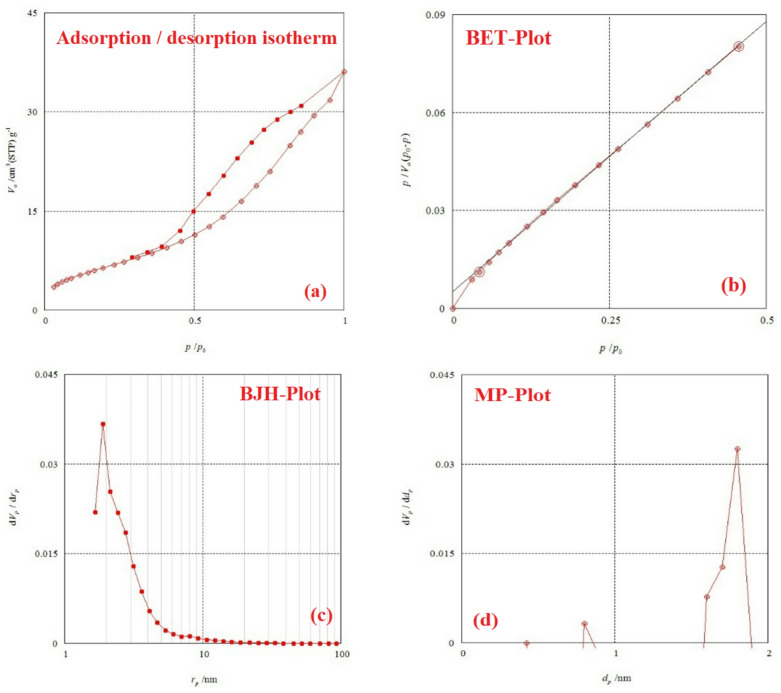
(**a**) Graph of N_2_ adsorption-desorption, (**b**) BET, (**c**) BJH, and (**d**) MP analysis of the MAPA.

The efficiency of materials such as sorbents, catalysts, and membranes strongly depends on their physicochemical properties, particularly surface area and porosity. These structural attributes in nanomaterials are commonly analyzed using gas adsorption techniques, with the Brunauer–Emmett–Teller (BET) model being one of the most established approaches^41^. In this work, the specific surface area of the amino polyacrylonitrile nanocomposite was determined by the BET method, employing nitrogen adsorption–desorption isotherms^42^. As shown in Fig. 4(a), the obtained isotherm exhibits a hysteresis loop characteristic of type IV behavior, which reflects the presence of mesopores. The mean pore diameter of the magnetic amino polyacrylonitrile nanocomposite was calculated as 8.5341 nm^43,44^. Typically, these isotherms are depicted by plotting the adsorbed gas volume as a function of the relative pressure (*p*/*p*°)^45^.

#### XRD study of MAPA

**Fig. 5:**
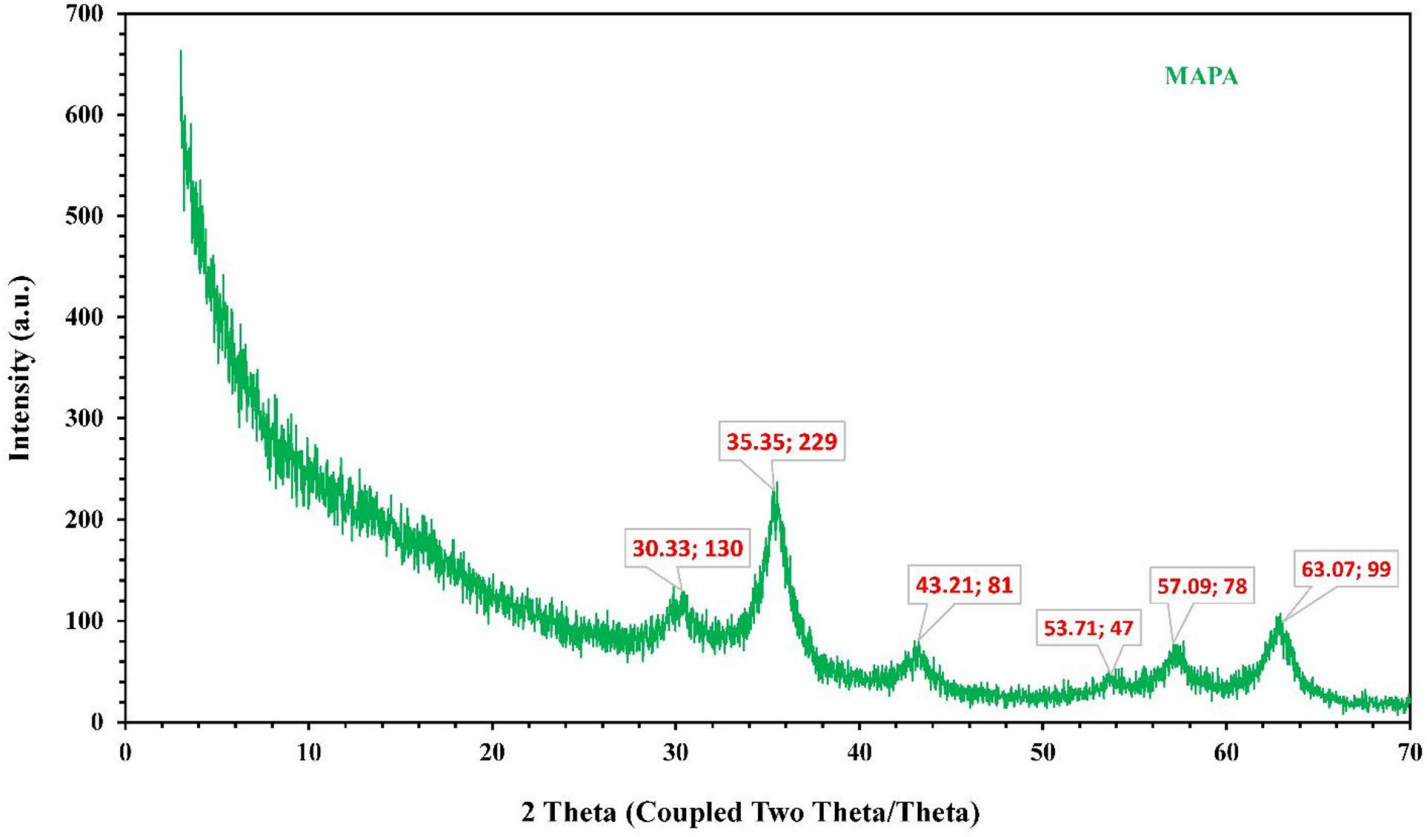
XRD graph of fabricated MAPA nanocomposite.

X-ray diffraction (XRD) serves as a key method for elucidating materials’ crystalline structures. The resulting XRD patterns act as distinctive “fingerprints,” providing insights into crystal phases, lattice parameters, and crystallite dimensions. In this study, the crystalline structure of the MAPA was examined by XRD, as depicted in Fig. 5. Measurements were performed over a 2*θ* range of 5°–70°. Prominent diffraction peaks observed at 2*θ* values of 30.33°, 35.35°, and 63.07° indicate the presence of magnetite nanoparticles exhibiting a spinel structure, consistent with JCPDS card No. 98-3969^46,47^. The incorporation of the MAPA polymer appears to have a negligible influence on the structural integrity of the magnetite nanoparticles.

#### Magnetic properties of MAPA nanocomposite

Analyzing data obtained from a Vibrating Sample Magnetometer (VSM) requires careful examination of several fundamental magnetic parameters. The VSM measures the magnetization (M) of a sample as a function of the applied magnetic field (H), generating a hysteresis loop that characterizes the material’s magnetic behavior^48,49^. This loop demonstrates the extent to which the sample retains magnetization after removing external field. One important parameter is coercivity, which defines the intensity of the magnetic field necessary to bring the magnetization to zero following saturation^48,49^. Coercivity is useful for differentiating between hard and soft magnetic materials: hard magnets display high coercivity and retain their magnetization effectively, whereas soft magnets exhibit low coercivity and are easily demagnetized. Saturation magnetization (Ms) represents the maximum magnetization achievable under a strong applied field. In contrast, remanent magnetization (Mr), or retentivity, quantifies the residual magnetization retained once the field is removed, reflecting the material’s ability to preserve magnetic properties. Additionally, the hysteresis loop’s shape provides further information on magnetic hardness, with narrow loops corresponding to soft materials and wide loops indicative of hard materials^48,49^.

**Fig. 6:**
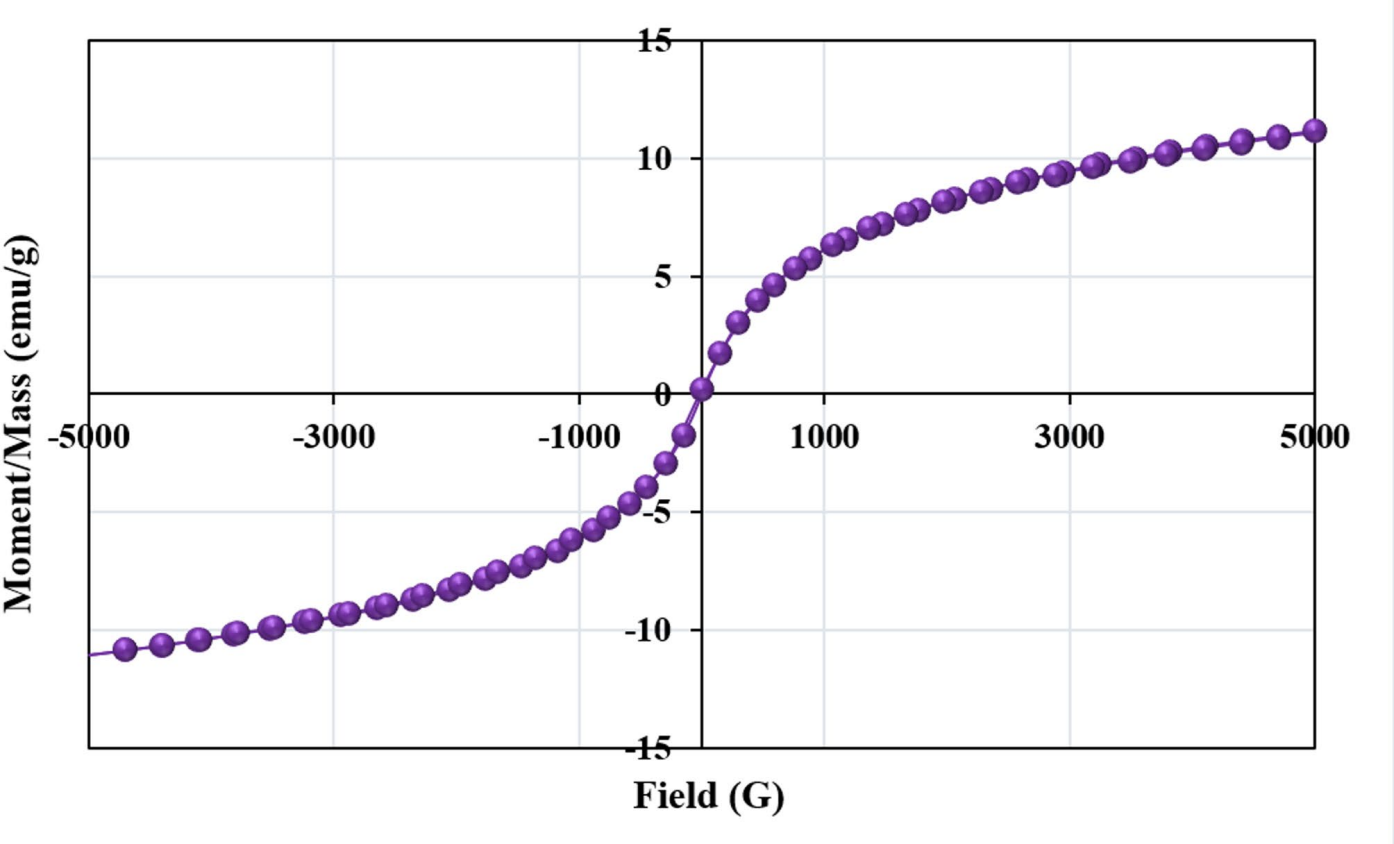
Magnetization curve for MAPA nanocomposite.

Vibrating Sample Magnetometer (VSM) measurements of the MAPA nanocomposite were performed under an applied magnetic field ranging from − 5000 to 5000 Oe. As shown in Fig. 6, the nanocomposite exhibited a saturation magnetization (Ms) of 11.098 emu/g, a coercivity of 9.9567 G, and a remanent magnetization (Mr) of 0.10541 emu/g. The observed S-shaped hysteresis loop, characterized by its considerable width and relatively high coercivity, confirms the nanocomposite’s classification as a hard magnetic material, indicating its suitability for magnetic separation after adsorption processes^48,49^.

#### X-ray photoelectron spectroscopy (XPS) of MAPA nanocomposite

**Fig. 7:**
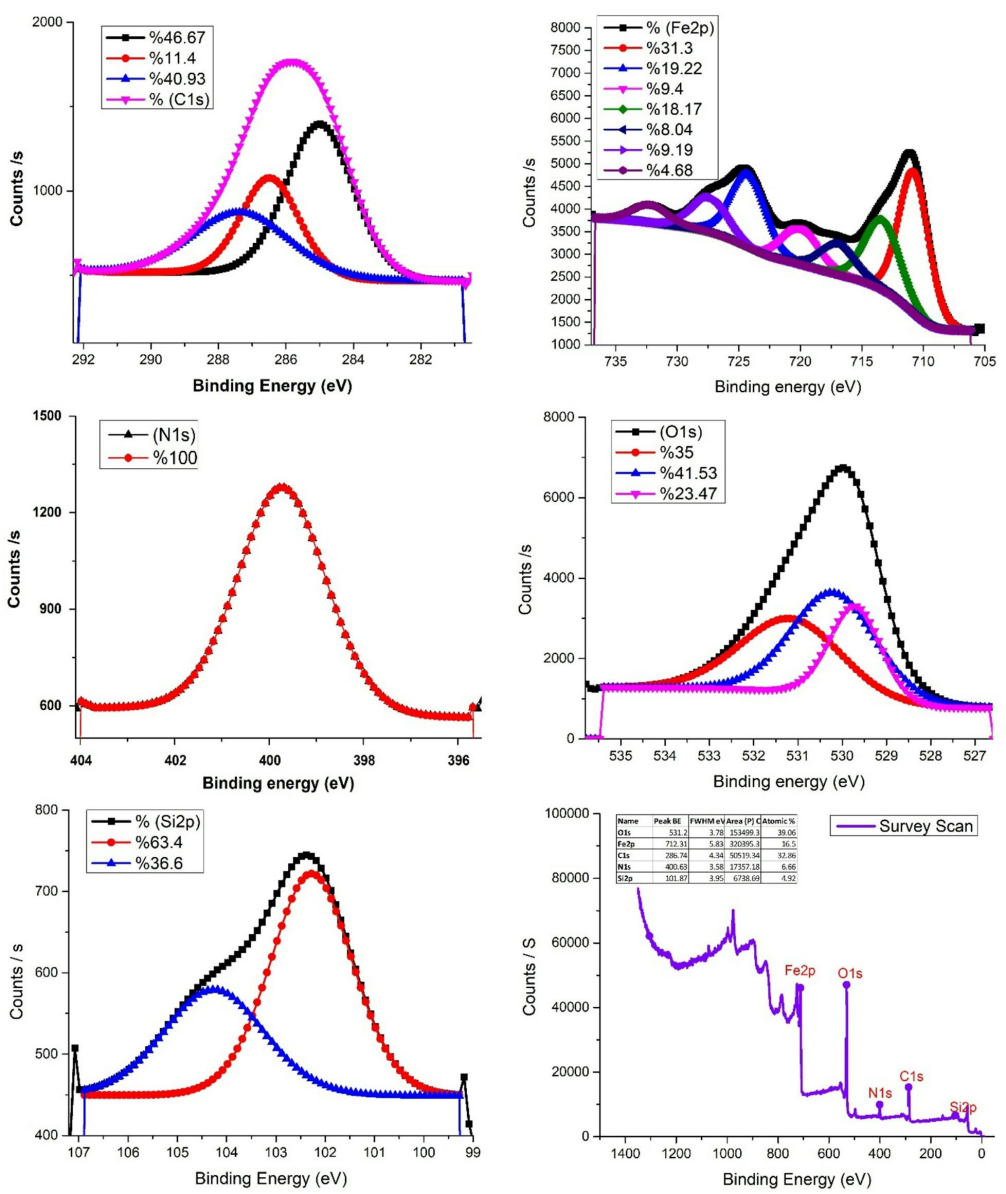
XPS spectrum of the MAPA nanocomposite adsorbent recorded at a resolution of 1 eV.

X-ray Photoelectron Spectroscopy (XPS) is a surface-sensitive and quantitative method widely employed to investigate materials’ elemental composition, oxidation states, and electronic environments^48,49^. In an XPS spectrum, the binding energy (eV) is plotted on the X-axis, reflecting the strength of electron binding to atoms, while the Y-axis denotes intensity, expressed as counts or arbitrary units, which corresponds to the number of electrons detected at each binding energy^48,49^. Figure 7 presents the XPS spectrum of the MAPA nanocomposite, confirming the presence of major constituent elements. Distinct peaks were identified at 286.74 eV (C 1s), 531.2 eV (O 1s), and 712.31 eV (Fe 2p). The C 1s peak at 286.74 eV arises from carbon atoms in the (C ≡ N) functional group, while the O 1s peak at 531.2 eV is associated with lattice oxygen in Fe₃O₄, verifying the presence of magnetite. The Fe 2p spectrum displayed characteristic signals at 704 eV, 721 eV, and 727.08 eV, corresponding to Fe 2p₃/₂, Fe 2p₁/₂, and an additional Fe 2p₃/₂ peak, further supporting the identification of magnetite. Moreover, a peak at 400.63 eV in the N 1s region indicates nitrogen incorporation within the nanocomposite structure.

### Adsorption investigation

This study employed the batch equilibrium technique to examine the adsorption behavior of copper ions (Cu^2+^) onto a novel material, Magnetic Amino Polyacrylonitrile (MAPA) Nanocomposite. The effect of key operational parameters, including contact time, solution pH, adsorbent dose, and initial Cu^2+^ concentration, was systematically evaluated to assess the adsorption process.

#### Effect of pH

**Fig. 8:**
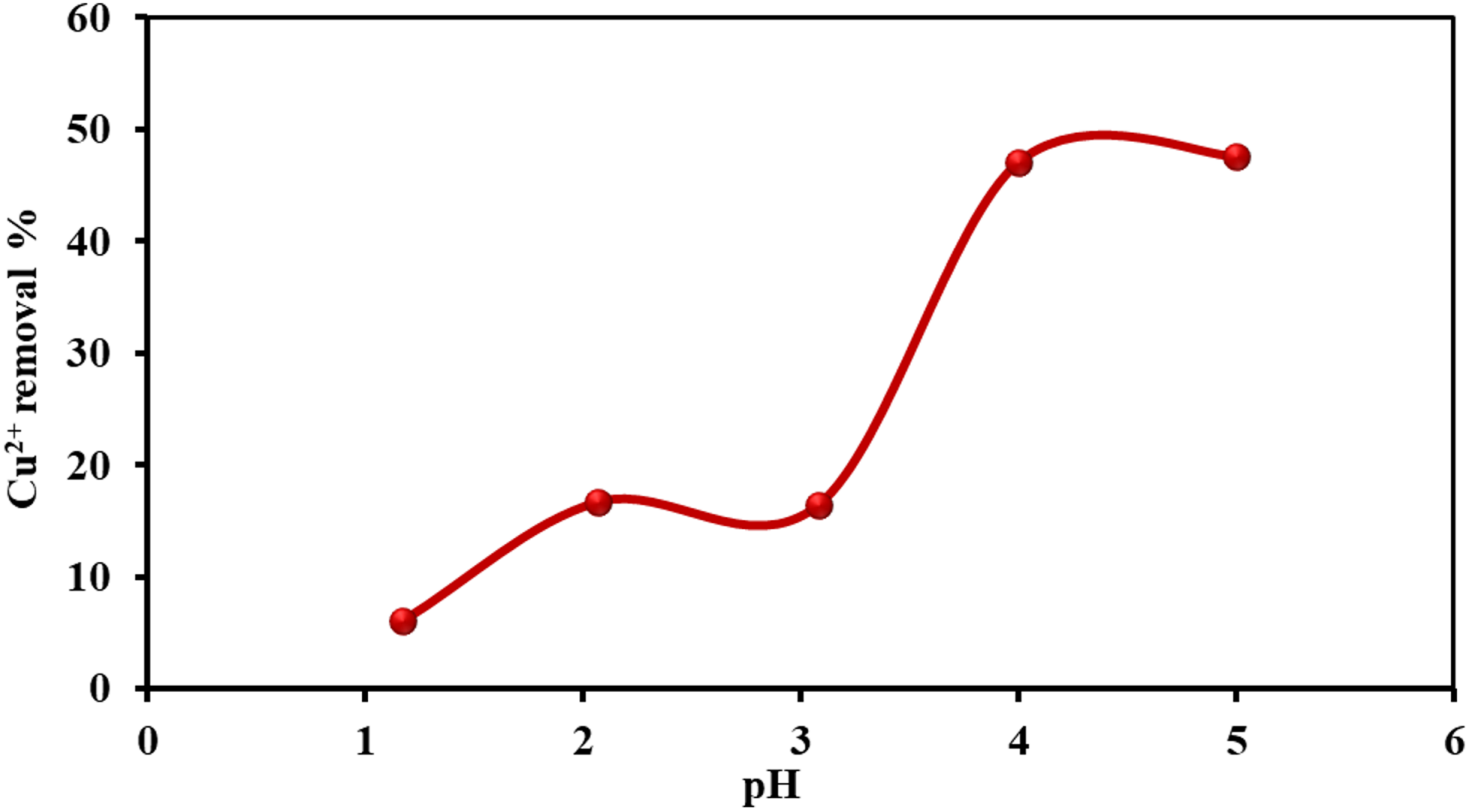
Effect of solution pH on the removal efficiency of Cu^2+^ ions by the MAPA adsorbent [Cu^2+^ (100 mg/L), MAPA (5.0 g/L), temp. (25 °C)].

The initial solution pH is a critical factor in the adsorption process, as it governs both the speciation of copper ions and the surface charge characteristics of the adsorbent^50^. This study investigated pH values between 1 and 6, since at pH levels higher than 6, Cu^2+^ ions tend to precipitate as Cu(OH)_2_, which could interfere with adsorption measurements^51^. As shown in Fig. 8, the adsorption of Cu^2+^ ions onto the MAPA nanocomposite was strongly dependent on pH. Adsorption efficiency gradually rose from pH 1 to 3.1, followed by a pronounced increase in copper uptake. Excess H^+^ ions reduced adsorption at lower pH conditions by competing with Cu^2+^ ions for available binding sites. Increasing pH diminished the H^+^ ion concentration, lowering competition and facilitating more effective copper adsorption.

#### Effect of MAPA nanocomposite adsorbent dosage

This experimental section focused on evaluating the influence of adsorbent dosage, specifically the amount of MAPA nanocomposite, on the removal efficiency of Cu^2+^ ions. The adsorption experiments were conducted under the following experimental conditions: initial Cu^2+^ ion concentrations ranging from 50 to 150 mg/L, adsorbent doses between 2.0 and 6.0 g/L, solution temperature maintained at 25 °C, contact time fixed at 60 min, and solution pH was adjusted to 5.5.

**Fig. 9:**
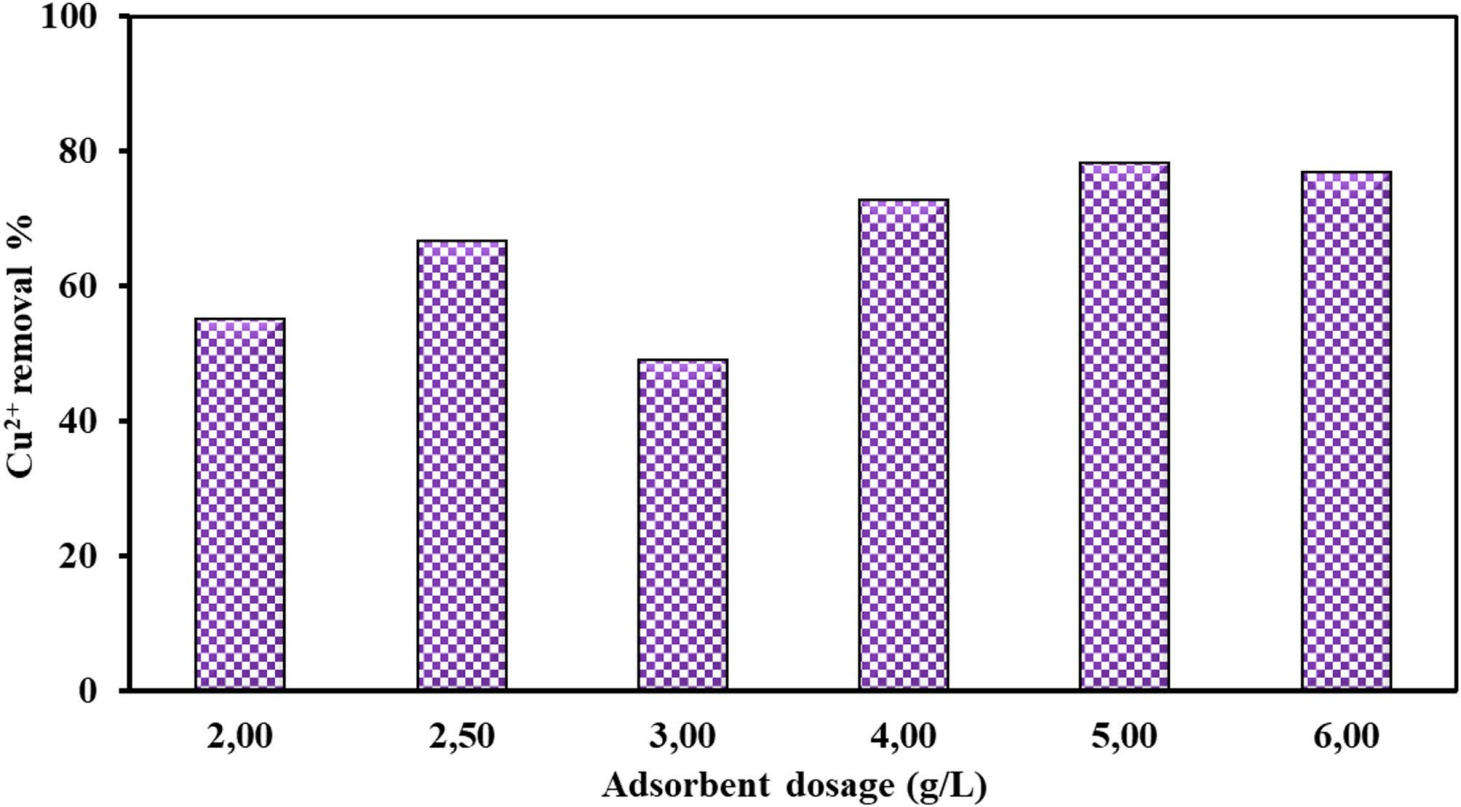
Effect of MAPA nanocomposite doses (2.0–6.0 g/L) on 50 mg/L Cu^2+^ ion concentration.

As illustrated in Fig. 9, an increase in adsorbent dosage improved the removal efficiency of Cu^2+^ ions. Specifically, when the concentration of MAPA nanocomposite was raised from 2.0 to 6.0 g/L, the Cu^2+^ ions removal rate increased from 55.1% to 76.8%. This improvement can be attributed to the greater availability of active binding sites on the adsorbent, while the total amount of Cu^2+^ ions in solution remained constant.

#### Effect of contact time

**Fig. 10:**
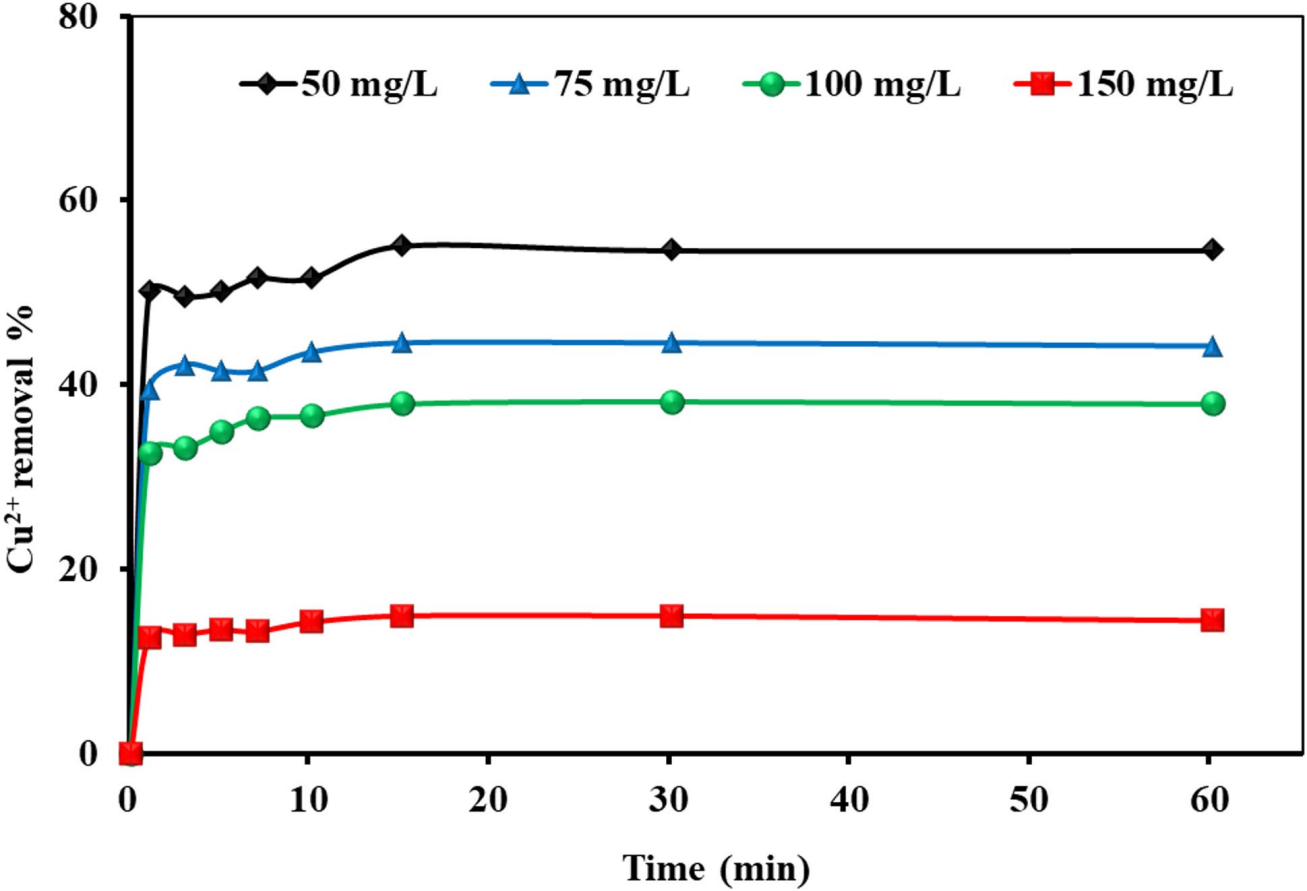
The Cu^2+^ ions removal % using MAPA as an adsorbent (Cu^2+^ ions = (50–150 mg/L), MAPA dose = 2.0 g/L, Temp. = 25 °C).

An experiment was performed to evaluate the influence of contact time on the adsorption of Cu^2+^ ions onto MAPA nanocomposite. In this study, an adsorbent dosage of 2.00 g/L was applied with initial Cu^2+^ concentrations ranging from 50 to 150 mg/L. As presented in Fig. 10, the adsorption occurred rapidly, with more than 50% of Cu^2+^ ions removed within the first few minutes. Equilibrium was reached in approximately 10 min, and the removal efficiency decreased as the initial ion concentration increased. The rapid uptake at the beginning is attributed to the abundance of available active sites on the adsorbent surface, while the subsequent slowdown reflects site saturation over time.

### Adsorption kinetics

Various kinetic models were applied to gain deeper insights into the adsorption mechanism and interpret the experimental data. Commonly employed in adsorption research, these models include the pseudo-first-order (PFO), pseudo-second-order (PSO), intraparticle diffusion (IPDM), and film diffusion (FDM) models^52,53^. In the present work, the adsorption of Cu^2+^ ions onto the magnetic amino polyacrylonitrile nanocomposite was assessed by fitting the obtained experimental data to each of these four models. The PFO can be expressed by Eq. (4):

4 $$\:\mathrm{log}\left({q}_{\mathrm{e}}-{q}_{\mathrm{e}}\right)=\mathrm{log}\left({q}_{\mathrm{e}}\right)-\frac{{k}_{1}}{2.303}t$$

where, *q*_t_ (mg/g) represents the amount of copper adsorbed onto the prepared nanocomposite at time *t* (min), *q*_*e*_ (mg/g) denotes the amount of copper adsorbed at equilibrium, and *k*_*1*_ (L/min) is the equilibrium rate constant for the PFO adsorption process.

**Fig. 11:**
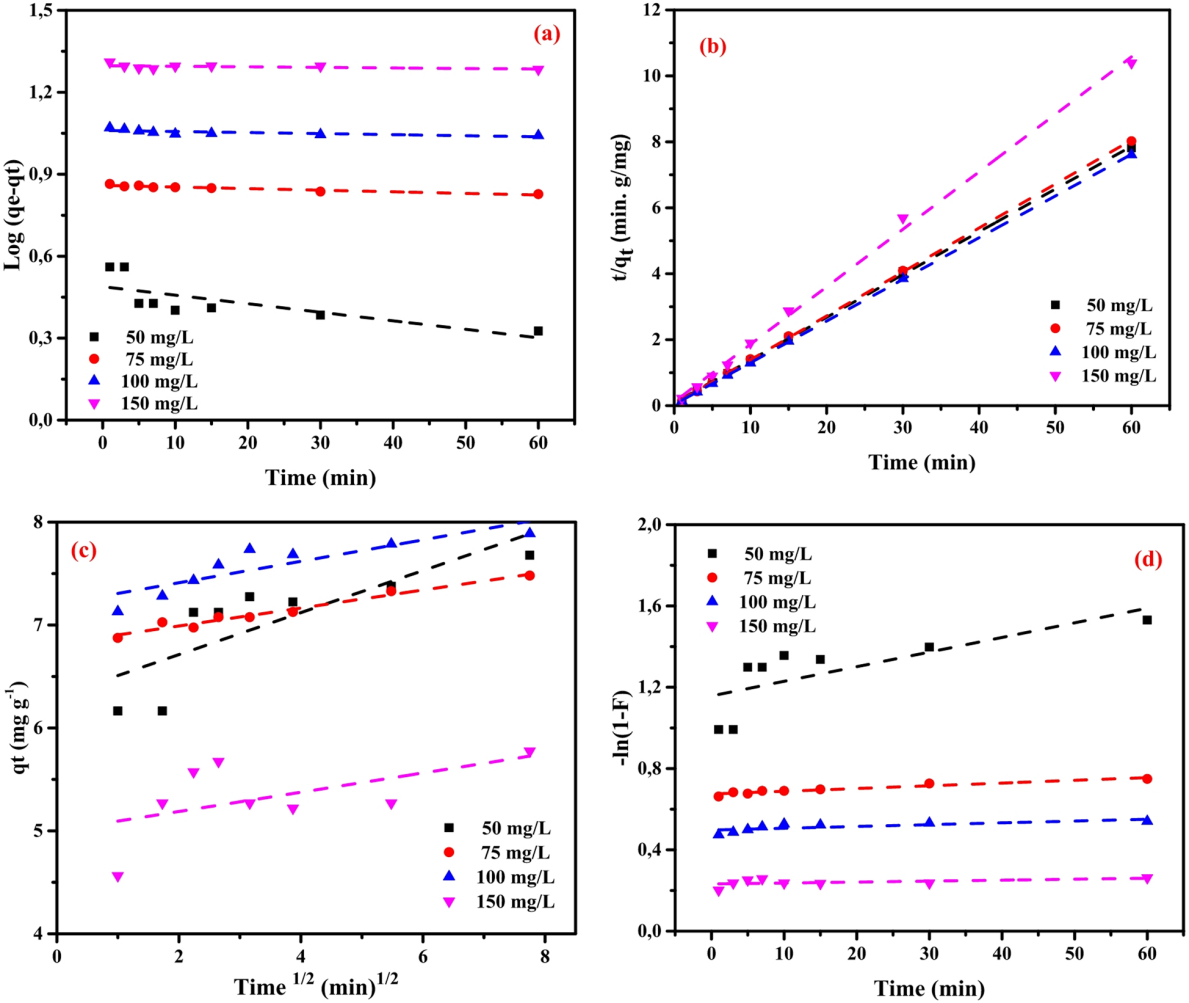
(**a**) PFO, (**b**) PSO, (**c**) IPDM, and (**d**) FDM kinetic models of adsorption of Cu^2+^ ions by MAPA nanocomposite (C_0_ = (50–150 mg/L), MAPA dose = (6.0 g/L), Temp. = 25 °C).

As illustrated in Fig. 11(a), the plot of log(*q*_*e*_ – *q*_*t*_) versus time (*t*) exhibits a linear trend, where the slope corresponds to the rate constant *k*_*1*_​ and the intercept represents the equilibrium adsorption capacity (*q*_*e*_​). Nevertheless, the experimental findings indicate that the PFO does not adequately describe the adsorption of Cu²⁺ ions onto the magnetic adsorbent. This inadequacy arises from the relatively low correlation coefficient and the considerable deviation between the calculated and experimental *q*_*e*_​ values. Consequently, the pseudo-second-order model (PSO), defined by Eq. (5), was evaluated as an alternative.

5 $$\:\left(\frac{t}{{q}_{t}}\right)=\frac{1}{{k}_{2}{{q}_{e}}^{2}}+\frac{1}{{q}_{e}}\left(t\right)$$

where, *q*_t_ (mg/g) represents the amount of copper adsorbed onto the prepared nanocomposite at a given time *t* (min), *q*_e_ (mg/g) denotes the adsorption capacity at adsorption equilibrium, and *k*_2_ (g/mg min) represents the kinetic rate constant for the PSO. The initial adsorption rate (h) was determined using Eq. (6):

6 $$\:h={k}_{2\:}{{q}_{\mathrm{e}}}^{2}$$

Table 3 demonstrates that the PSO provides a significantly better representation of Cu^2+^ ion adsorption onto the MAPA nanocomposite compared with the PFO. The PSO consistently yields higher correlation coefficients (*R*^2^) and calculated adsorption capacities that closely align with the experimental values. These results indicate that the PSO provides the most reliable description of the adsorption behavior in this system.

Kargi and Cikla^54^ investigated the mass transfer mechanisms between the liquid phase and the adsorption of target pollutants onto the MAPA adsorbent^54^. Isothermal plug flow diffusion (IPDM) could act as the rate-limiting mechanism in a batch experimental setup under conditions of vigorous agitation^55^. The IPDM analysis was further conducted using Eq. 7, following the approach proposed by Annadurai et al.^56^.

7 $$\:{q}_{t}={K}_{diff}{t}^{0.5}+C$$

where *K*_*diff*_ represents the rate constant of IPDM (mg g^− 1^ min^1/2^) (Fig. 11c).

Weber and Morris^57^ propose that the adsorption process is dictated by the intraparticle diffusion step, as seen by the intersection of the lines indicating *q*_t_ and the square root of time (*t*) at the vector origin in Fig. 11c. In instances when the lines do not intersect the origin, it is generally acknowledged that the elimination process is predominantly influenced by the Film Diffusion Model (FDM), particularly when the *C* value is significantly high. The study investigated the efficiency of Cu²⁺ ion removal from aqueous solutions by varying the adsorbent dosage and the initial Cu²⁺ ion concentration. Figure 11c depicts the Webber-Morris^57^ adsorption plots for Cu^2+^ ions. The *K*_dif_ and *C* values presented in Table 4 were derived from the analysis of the slope and intercept of the plot illustrating the relationship between *q*_t_ and *t*^0.5^. The linear trends shown in Fig. 11c, representing different concentrations of the adsorbent, do not meet at the origin of Cu^2+^ ions. This can be ascribed to the noted elevated *C* intersection. This claim is supported by the observation that FDM markedly affects the absorption rate of Cu^2+^ ions into the MAPA adsorbent. The measured absorption rate shows a gradual increase over time, as illustrated in Fig. 11c. The intra-particle diffusion rate constant, *K*_dif_, ranged from 0.03 to 1.66 mg g^–1^min^–1/2^ for the loading of Cu^2+^ ions onto MAPA. The rate constant exhibited an increasing trend when the starting concentration of Cu^2+^ ions rose, concurrently with a reduction in the dosage of MAPA. The observed phenomena can be ascribed to the progressive decrease in the pore volume and surface area of the MAPA adsorbent throughout the separation process.

The film diffusion model (FDM) denotes the mechanism by which adsorbate molecules traverse a liquid film around the adsorbent particle. The equation for FDM is designated as Eq. 8.

8 $$\:\mathrm{ln}\left(1-F\right)={K}_{FD}\left(t\right)$$

where *K*_FD_ denotes the external film mass transfer coefficient and *F* represents defined as the ratio of *q*_t_ to *q*_e_.

The *K*_FD_ constant can be calculated from the slope and intercept of the plot of the logarithm of (1 − *F*) in relation to the plots (Fig. 11d)^58^. The PSO was identified as the most appropriate kinetic model for the electrochemical elimination of Cu^2+^ ions on MAPA. The aforementioned result was drawn from the observation that the straight lines did not overlap at the sources. The results suggest that film diffusion does not constitute the rate-limiting step in the overall adsorption kinetics. PSO rate data had the highest coefficient of determination (*R*^2^ = 1). The initial phase of the process is proposed to involve electrostatic interactions between the negatively charged active sites on the self-doped biochar carbon and the hydrogen ions in solution. This hypothesis is supported by the adsorption isotherm models PSO, LIM, and TIM. The specified compound comprised many nitrogen atoms that displayed unshared electron pairs. The surface of the MAPA, after positive self-doping, demonstrated effective absorption of Cu^2+^ ions, creating a unique adsorption layer. The presence of –NH_2_ group in the prepared MAPA is the essential group for the removal of Cu^2+^ ions via electrostatic attraction.

**Table 3:** PFO and PSO kinetic model results of adsorption of Cu^2+^ ions by MAPA nanocomposite adsorbent [Initial concentration (50–150 mg/L), MAPA nanocomposite doses (2.0–6.0 g/L), Temp. (25 °C)].

Parameter			PFO			PSO			
MAPA conc. (g/L)	Cu^2+^ conc. (mg/L)	q_e_ (exp.)	q_e_ (calc.)	k_1_ × 10^3^	*R* ^2^	q_e_ (calc.)	k_2_ × 10^3^	h	*R* ^2^
2.0	50	13.77	0.58	12.67	0.299	13.74	225.53	42.55	1.000
	75	16.68	10.50	2.07	0.427	16.64	298.51	82.65	1.000
	100	28.93	14.47	38.69	0.859	28.49	7.62	6.18	0.943
	150	11.16	22.11	0.92	0.377	16.64	2.69	0.744	1.000
2.5	50	13.34	13.131	3.4545	0.848	13.46	71.23	12.90	0.999
	75	13.45	16.604	1.1515	0.459	13.42	344.74	62.11	1.000
	100	15.37	10.397	2.7636	0.437	15.29	262.40	61.35	1.000
	150	9.23	26.194	0.4606	0.330	8.82	1368.04	106.38	0.999
3.0	50	8.17	8.833	1.15	0.766	8.13	179.25	11.85	0.998
	75	11.29	11.238	1.61	0.803	11.34	189.74	24.39	1.000
	100	13.06	8.636	2.76	0.659	13.02	124.96	21.19	0.999
	150	7.94	18.750	0.46	0.185	7.37	928.65	50.51	0.998
4.0	50	9.09	4.501	7.60	0.779	9.20	124.64	10.55	1.000
	75	8.72	9.432	1.84	0.489	8.72	282.32	21.46	1.000
	100	9.86	9.892	1.61	0.708	9.82	170.17	16.42	0.999
	150	6.34	17.262	0.02	0.000	5.98	476.25	17.04	0.999
5.0	50	7.83	3.099	9.44	0.681	7.90	150.63	9.40	1.000
	75	6.93	7.268	0.23	0.244	6.93	2665.85	128.21	1.000
	100	8.95	10.382	2.99	0.929	8.90	70.49	5.59	0.993
	150	5.88	17.282	0.46	0.123	5.35	742.43	21.28	0.997
6.0	50	7.68	3.079	7.14	0.567	7.71	199.55	11.86	1.000
	75	7.48	7.228	1.38	0.916	7.50	316.74	17.83	1.000
	100	7.79	11.487	0.92	0.578	7.91	442.58	27.70	1.000
	150	5.78	19.824	0.46	0.246	5.73	262.65	8.64	0.998

**Table 4:** IPDM and FDM kinetic model results of Cu^2+^ ions adsorption by MAPA nanocomposite adsorbent [Initial concentration (50–150 mg/L), adsorbent doses (2.0–6.0 g/L), Temp. (25 °C)].

Parameter			IPDM			FDM		
MAPA conc. (g/L)	Cu^2+^ conc. (mg/L)	q_e_ (exp.)	K_dif_	C	*R* ^2^	K_FD_	C	*R* ^2^
2.0	50	13.77	0.22	12.27	0.695	0.0039	0.75	0.546
	75	16.68	0.24	15.15	0.610	0.0022	0.91	0.427
	100	28.93	1.66	13.45	0.813	0.0090	0.92	0.704
	150	6.34	0.24	9.51	0.579	0.0010	0.38	0.377
2.5	50	13.34	0.41	10.39	0.941	0.0035	0.61	0.848
	75	13.45	0.20	12.17	0.651	0.0011	0.56	0.459
	100	15.37	0.30	13.43	0.642	0.0027	0.89	0.437
	150	9.23	0.10	8.23	0.489	0.0004	0.28	0.330
3.0	50	8.17	0.09	7.29	0.636	0.0013	0.61	0.766
	75	11.29	0.16	10.12	0.883	0.0016	0.66	0.803
	100	13.06	0.21	11.37	0.717	0.0027	0.86	0.659
	150	7.94	0.12	6.85	0.368	0.0005	0.32	0.185
4.0	50	9.09	0.28	7.16	0.889	0.0075	1.00	0.779
	75	8.72	0.16	7.62	0.610	0.0010	0.61	0.489
	100	9.86	0.14	8.68	0.755	0.0016	0.64	0.708
	150	6.34	0.03	6.00	0.038	0.0000	0.30	0.000
5.0	50	7.83	0.24	6.18	0.745	0.0094	1.15	0.681
	75	6.93	0.02	6.78	0.345	0.0003	0.66	0.244
	100	8.95	0.24	6.73	0.858	0.0030	0.52	0.929
	150	5.88	0.11	4.88	0.289	0.0005	0.26	0.123
6.0	50	7.68	0.20	6.30	0.657	0.0072	1.16	0.567
	75	7.48	0.09	6.82	0.967	0.0013	0.67	0.916
	100	7.79	0.10	7.20	0.761	0.0009	0.50	0.578
	150	5.78	0.09	5.00	0.302	0.0005	0.23	0.246

### Adsorption isotherms

Adsorption isotherms play a crucial role in elucidating the mechanisms governing adsorbent performance. They provide a framework for interpreting the equilibrium relationship between an adsorbate (the substance retained) and an adsorbent (the material responsible for retention). Examining these models allows determination of the maximum adsorption capacity, an important factor in optimizing the applicability of the adsorbent. Moreover, the data reveal valuable information about molecular interactions and binding affinities^59,60^.

In this study, three widely applied isotherm models—Langmuir (LIM) (Eq. 9), Freundlich (FIM) (Eq. 8), and Temkin (TIM) (Eq. 9)—were employed to evaluate the adsorption of Cu²⁺ ions onto a magnetic amino polyacrylonitrile (MAPA) nanocomposite, aiming to gain deeper insight into the adsorption mechanism and the ion–surface interactions involved. Based on these assumptions these assumptions, the model yields a characteristic linear equation that quantitatively represents adsorption behavior^59,60^.

9 $$\:\frac{{\mathrm{C}}_{e}}{{\mathrm{q}}_{e}}=\frac{1}{{\mathrm{K}}_{L}{\mathrm{q}}_{m}}+\frac{1}{{\mathrm{q}}_{m}}\times\:{\mathrm{C}}_{e}$$

where, *C*_e_ is the concentration of adsorbate in solution at equilibrium, *q*_m_ (mg/g) represents the theoretical maximum adsorption capacity, *q*_e_ (mg/g) indicates the equilibrium adsorption capacity, and *K*_L_ represents the Langmuir constant (L/mg) associated with adsorption energy^59,60^.

The Freundlich isotherm model characterizes adsorption behavior at equilibrium by expressing the relationship between the quantity of adsorbate retained per unit mass of adsorbent and the equilibrium concentration of the adsorbate remaining in solution^59,60^.

10 $$\:\mathrm{log}q\mathrm{e}=\mathrm{log}K\mathrm{F}\:+\:\frac{1}{n}\:\mathrm{log}C\mathrm{e}$$

where, *n* represents a constant related to the relation between the adsorbate and adsorbent, *K*_F_ (mg/g) denotes the Freundlich constant reflecting adsorption capacity, *q*_e_ (mg/g) denotes the amount of Cu^2+^ ions removed per gram adsorbent at equilibrium, and *C*_e_ (mg/L) signifies the equilibrium concentration of Cu^2+^ ions in the solution.

The Temkin isotherm model (TIM) describes adsorption based on the assumption of indirect interactions between adsorbate and adsorbent molecules^59^. According to this theory, the heat of adsorption decreases linearly with increasing surface coverage due to adsorbate–adsorbent interactions. The model assumes a uniform distribution of binding energies up to a maximum value. The Temkin isotherm can be mathematically represented by Eq. (11), which is often expressed in a simplified linear form as shown in Eq. (12)^59^.

11 $$\:{q}_{e}=\frac{RT}{{B}_{T}}\mathrm{l}\mathrm{n}\left({A}_{T}{C}_{e}\right)$$

12 $$\:{q}_{e}=\frac{RT}{{B}_{T}}\mathrm{ln}\left({A}_{T}\right)+\frac{RT}{{B}_{T}}\mathrm{l}\mathrm{n}\left({C}_{e}\right)$$

where *A*_T_ (L/mg) is the Tempkin isotherm constant, *B*_T_ (J g/mol mg) represents the adsorption energy (heat of adsorption) variation factor, *R* (8.314 J/mol K) represents the universal gas constant, and *T* (K) represents the absolute temperature.

A newly developed MAPA nanocomposite was evaluated for its efficiency in removing Cu^2+^ ions from aqueous solution. The adsorption data, summarized in Table 5, were interpreted using these three isotherm models. The Langmuir model provided an excellent fit, exhibiting a correlation coefficient greater than 0.962, whereas the Freundlich model yielded a relatively poor correlation of 0.418 at an adsorbent dosage of 2 g/L.

**Fig. 12:**
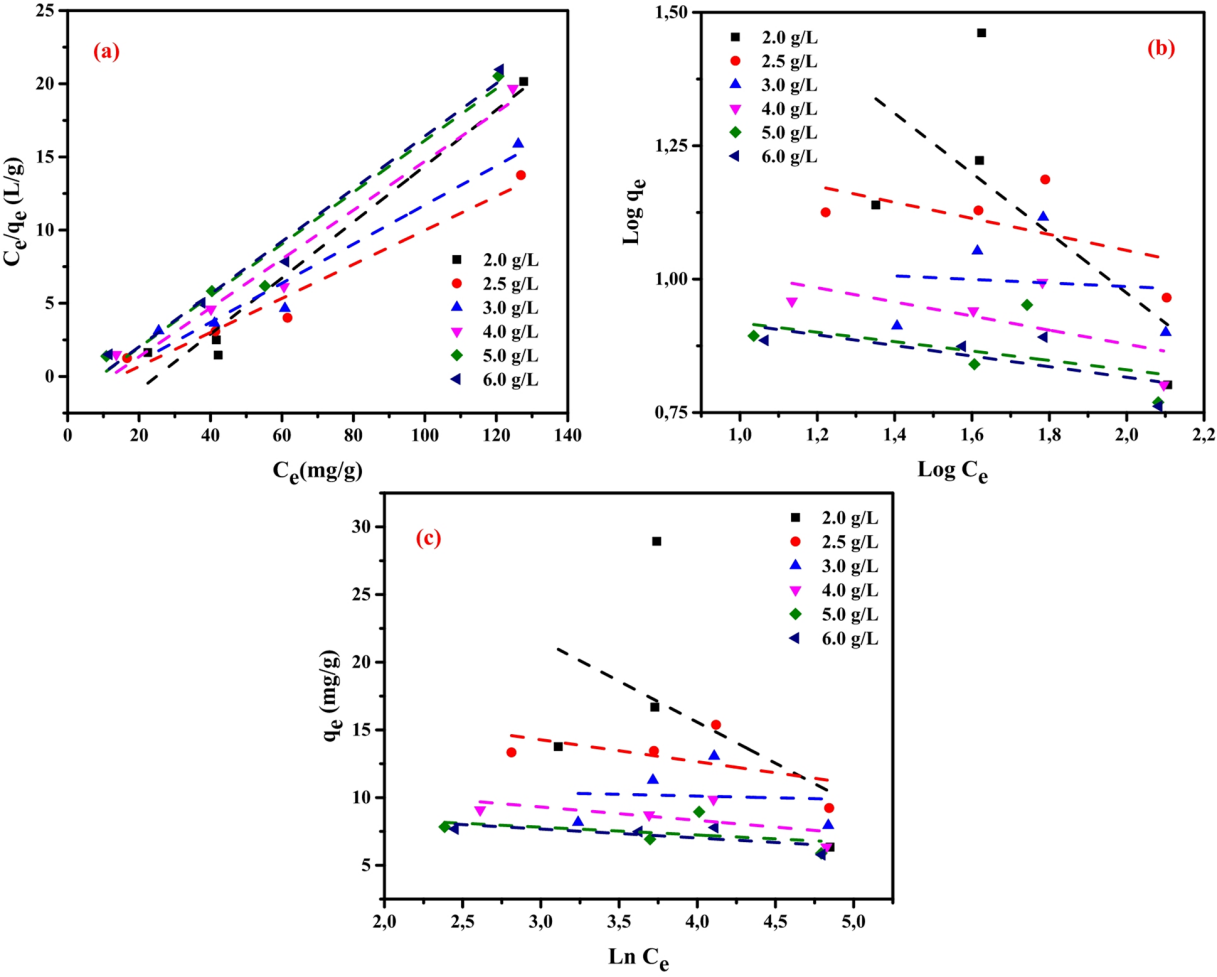
Isotherm profiles for Cu^2+^ ions on MAPA nanocomposite at initial concentrations of 50–150 mg/L and adsorbent dosages of 2.0–6.0 g/L at 25 °C: (**a**) LIM, (**b**) FIM, and (**c**) TIM.

The superior agreement with the Langmuir model indicates that the nanocomposite possesses a homogeneous surface, implying that all adsorption sites are structurally uniform and exhibit equal affinity toward Cu²⁺ ions (Fig. 12).

**Table 5:** Adsorption isotherm data for Cu^2+^ ions onto the MAPA nanocomposite adsorbent [Cu^2+^ (50–150 mg/L), adsorbent doses (2.0–6.0 g/L), temperature (25 °C)].

Isotherm model	Parameters	MAPA nanocomposite doses (g/L)					
		2.0	2.5	3.0	4.0	5.0	6.0
LIM	*q*_*m*_ (mg/g)	5.23	8.59	7.49	5.99	5.65	5.56
	*K*_*L*_ x10^3^	40.22	70.51	81.22	83.72	113.29	114.67
	*R* ^*2*^	0.966	0.962	0.949	0.968	0.972	0.981
FIM	*1/n*	0.56	0.15	0.03	0.13	0.09	0.10
	*K*_*F*_ (mg^1 − 1/n^ L^1/n^ g^–1^)	124.80	22.62	11.29	13.87	10.14	10.34
	*R* ^*2*^	0.418	0.338	0.009	0.396	0.243	0.482
TIM	*A*_*T*_	6.57	11.77	43.30	12.42	16.51	14.61
	*B*_*T*_	6.061	1.627	0.257	0.988	0.578	0.661
	*R* ^*2*^	0.218	0.284	0.005	0.361	0.197	0.477

### Comparison with results reported in the literature

The literature review in Table 6 compares the efficacy of other adsorbents in removing Cu^2+^ ions with that of the MAPA nanocomposite adsorbent, demonstrating that the MAPA adsorbent is effective.

**Table 6:** Comparative analysis of the maximum adsorption capacities of Cu^2+^ ions for various adsorbents.

Adsorbent	Max. capacity (mg/g)	Ref.
Sawdust	1.79	^61^
Saccharomyces cerevisiae	1.90	^62^
Orange peel	3.65	^63^
Tea fungal biomass	4.64	^64^
Datura innoxia	7.20	^65^
Banana peel	8.24	^66^
Tree fern	11.7	^67^
Synthetichematite (α-Fe_2_O_3_)iron oxide-coated sand	3.93	^68^
NaOH-treated rice husk	3.75	^69^
Magnetite Nano‑Adsorbent from Mill Scale Waste	4.42	^70^
Halloysite clay	3.42	^71^
MAPA	5.65	This work

### ANN modelling

**Fig. 13:**
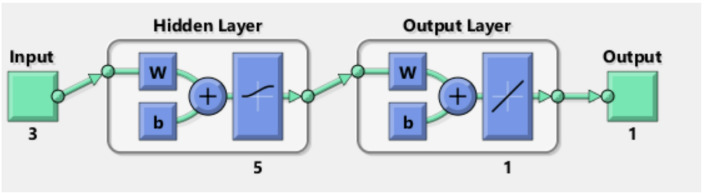
ANN architecture for the elimination of Cu^2+^ heavy metal.

**Fig. 14:**
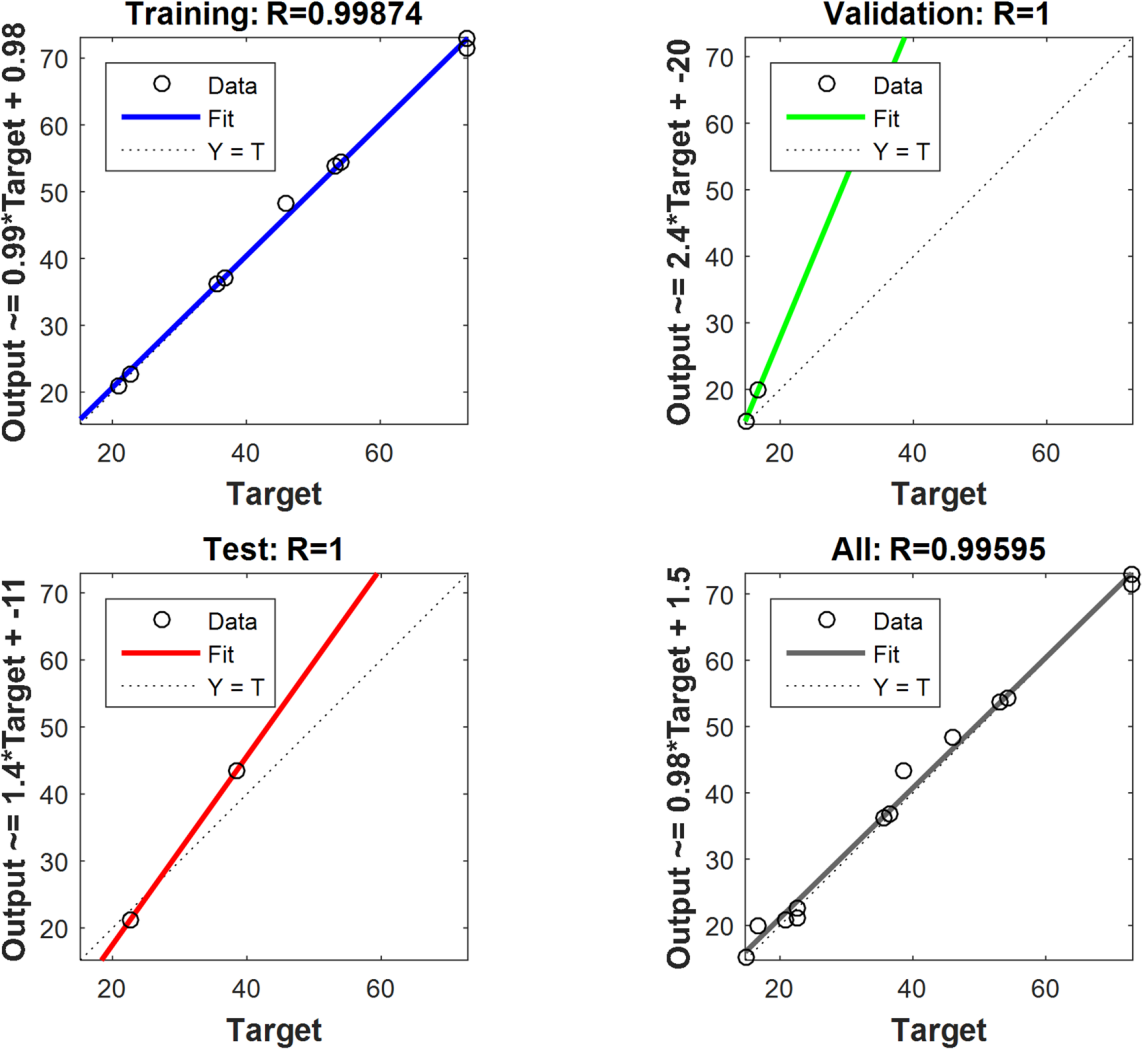
Training, validation, testing, and overall datasets for the LM algorithm.

**Fig. 15:**
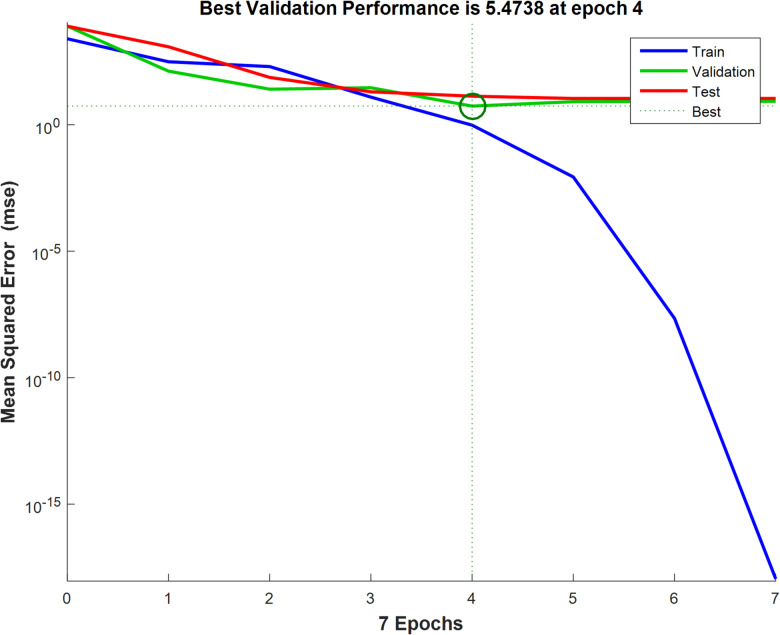
LM algorithm performance.

The backpropagation algorithm with sample data divided into training (70%), testing (15%), and Validation (15%) trained the optimal ANN model for removing Cu (II) by the MAPA which is formed of 3 neurons in the input layer, 5 neurons in the hidden layer, and 1 neuron in the output layer, as shown in Fig. 13. The regression plot illustrated that *R*² training was 0.99874. *R*^2^ validation and testing were 1, while the overall *R*² was 0.996 between the experimental results and the ANN predicted data. The regression plots are illustrated in Fig. 14. The MSE was determined to be 1.11e-28. The hidden layer employed the log-sigmoid (log-sig) activation function, while the output layer utilized the pure linear (purelin) activation function. The optimal ANN input variables are the adsorbent dosage of MAPA (g/L), time (min), and initial concentrations of Cu (II), while the output variable was the removal % of Cu (II). Figure 15 displayed the MSE error vs. the epoch number for the optimized ANN model that stopped after 4 epochs with the best validation of 5.4738^72,73^.

### RSM study

The chosen model was subjected to an ANOVA analysis to assess its relevance and identify the variables influencing the elimination percentage^74–81^. The experimental and anticipated elimination percentages and the ANOVA analysis outcomes are shown in Tables 1 and 7. The F-values show the significance of the variables and how they interact with the answer. A substantial impact of the factor or interaction on the answer is indicated by an F-value larger than 1. The Cu^2+^ removal % was most significantly impacted by the starting Cu^2+^ ion concentration, as Table 7 demonstrates. The model’s Adeq Precision-value of 176.21 (Table 7) emphasises its relevance.

Furthermore, factors are deemed significant if their p-values are less than 0.05. The relevance of the model is further supported by its p-value, which is less than 0.0001. The minor discrepancy between the adjusted *R*^2^ (0.9994) and predicted *R*^2^ (0.9957), which is less than 0.2, further demonstrates the robustness of the model. The following Eqs. (13, 14) for Cu^2+^ ion removal percentage were derived from the findings obtained:

13 $$\begin{aligned} {\text{Removal }}\% {\text{ for coded factors }} = & {\mathrm{36}}.{\text{66 }} + {\text{ 5}}.{\text{43A }} + {\text{ 6}}.{\text{71B }}{-}{\text{ 22}}.{\text{27C }}{-}{\text{ 1}}.{\text{39AB }}{-}{\text{ 3}}.{\text{68AC }} \\ & {-}{\text{ 2}}.{\text{96BC }}{-}{\text{ }}0.{\mathrm{29A}}^{2} {\text{ }}{-}{\text{ }}0.{\mathrm{71B}}^{2} {\text{ }} + {\text{ 4}}.{\mathrm{88C}}^{2} \\ \end{aligned}$$

14 $$\begin{aligned} {\text{Removal }}\% {\text{ for actual factors }} = & {\text{ 7}}.{\text{32 }} + {\text{ 7}}.{\text{69 Dose }} + {\text{ }}0.{\text{57 Time }}{-}{\text{ }}0.{\text{63 Cu dose }}{-}{\text{ }}0.0{\text{24 }}\left( {{\mathrm{Dose}}*{\mathrm{Time}}} \right){\text{ }} \\ & {-}{\text{ }}0.0{\text{368 }}\left( {{\mathrm{Dose}}*{\text{ Cu Conc}}.} \right){\text{ }}{-}{\text{ }}0.00{\mathrm{2}}0{\text{ }}\left( {{\mathrm{Time}}*{\text{Cu Conc}}} \right){\text{ }} \\ & {-}{\text{ }}0.0{\text{729 Dose}}^{2} {\text{ }}{-}{\text{ }}0.000{\text{81 Time}}^{2} {\text{ }} + {\text{ }}0.00{\text{195 Cu Conc}}^{2} \\ \end{aligned}$$

**Table 7:** *ANOVA* results for the key variables affecting Cu^2+^ ion removal, as determined by the Box–Behnken design.

Source	Sum of squares	df	Mean square	F-value	*p*-value	
Model	4762.04	9	529.12	2877.51	< 0.0001	Significant
A-MAPA dose	235.85	1	235.85	1282.61	< 0.0001	
B-Time	359.66	1	359.66	1955.93	< 0.0001	
C-Cu dose	3968.38	1	3968.38	21581.41	< 0.0001	
AB	7.70	1	7.70	41.88	0.0003	
AC	54.07	1	54.07	294.07	< 0.0001	
BC	35.11	1	35.11	190.92	< 0.0001	
A²	0.3581	1	0.3581	1.95	0.2055	
B²	2.10	1	2.10	11.41	0.0118	
C²	100.41	1	100.41	546.06	< 0.0001	
Residual	1.29	7	0.1839			
Lack of fit	1.29	3	0.4291			
Pure error	0.0000	4	0.0000			
Cor total	4763.33	16				
Std. dev.	0.4288	R²	0.9997			
Mean	38.49	Adjusted R²	0.9994			
C.V. %	1.11	Predicted R²	0.9957			
Adeq precision	176.2150					

**Fig. 16:**
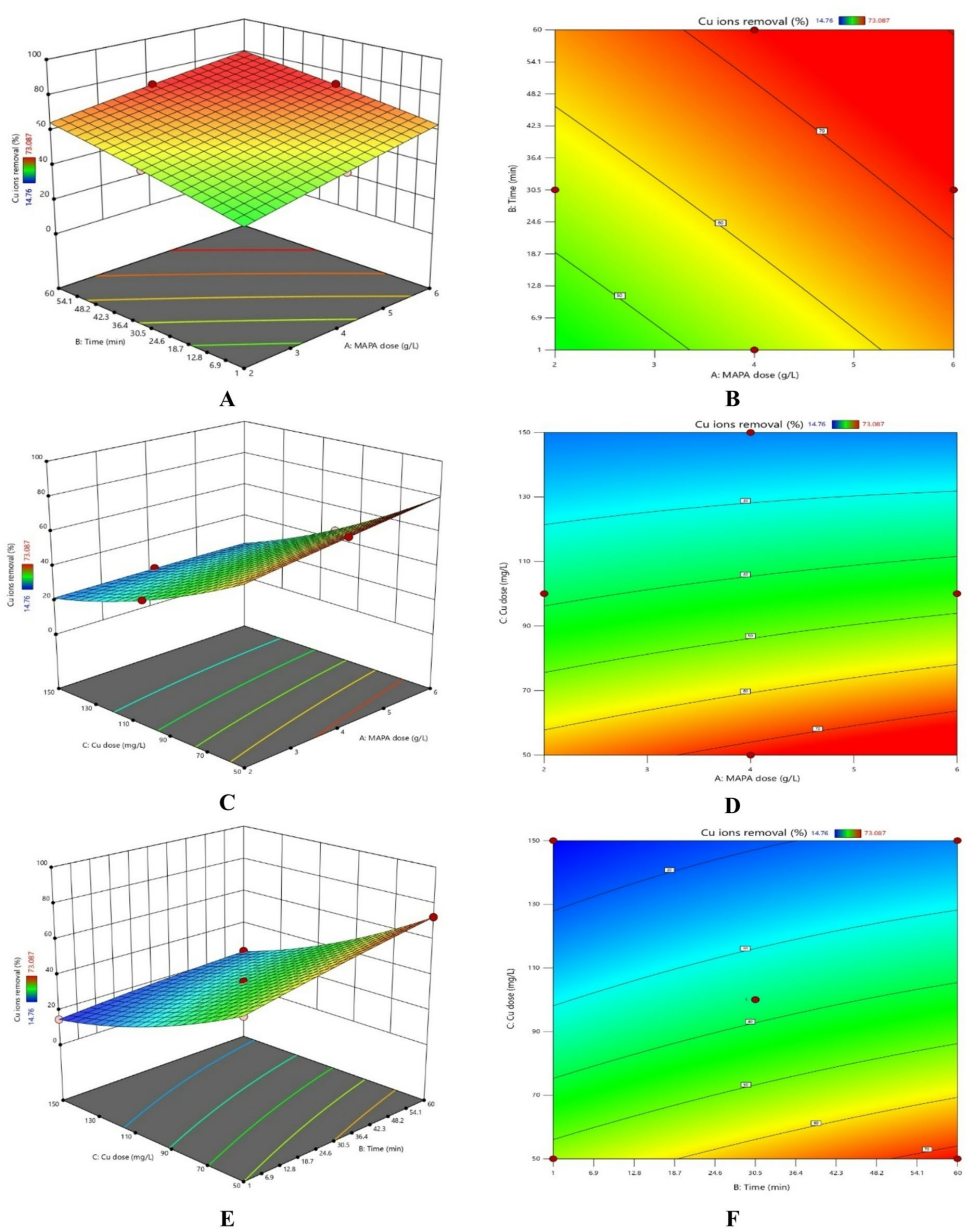
Combined effects of independent variables: (**A,B**) Adsorbent dose and time, (**C,D**) MAPA Adsorbent dose and Cu^2+^ initial concentration, and (**E,F**) Cu^2+^ initial concentration and time.

Figure 16 illustrates the combined influence of reaction time, adsorbent dosage, and initial Cu^2+^ concentration on the removal efficiency of Cu^2+^ ions. Low Cu^2+^ concentrations, large MAPA adsorbent doses, and prolonged reaction durations all contribute to the best removal percentages^79–81^.

**Fig. 17:**
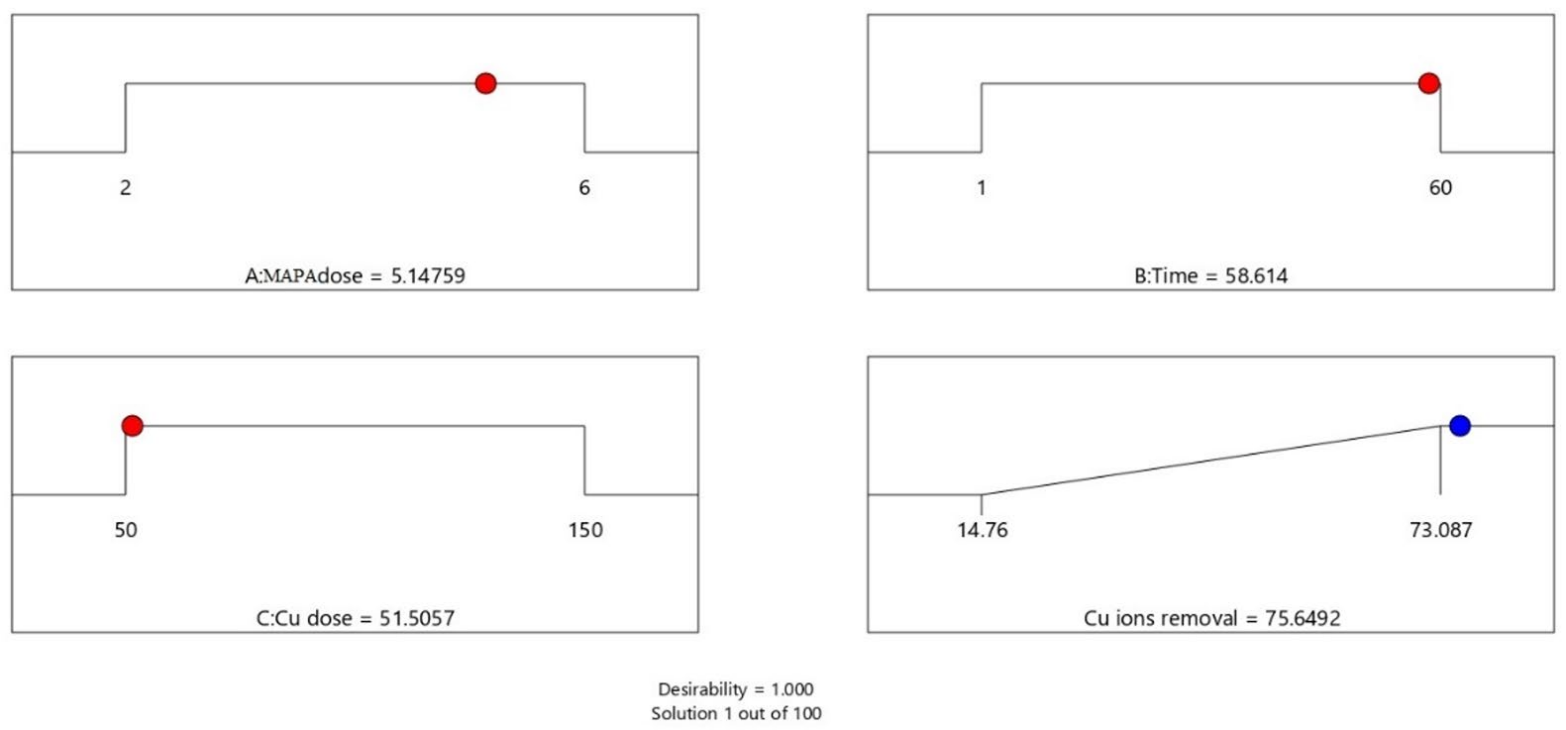
Optimization conditions through BBD settings.

The best operating settings to attain the maximum Cu^2+^ removal % were found statistically, as shown in Fig. 17.

### Mechanism of Cu^2+^ ions adsorption by MPAP

**Fig. 18:**
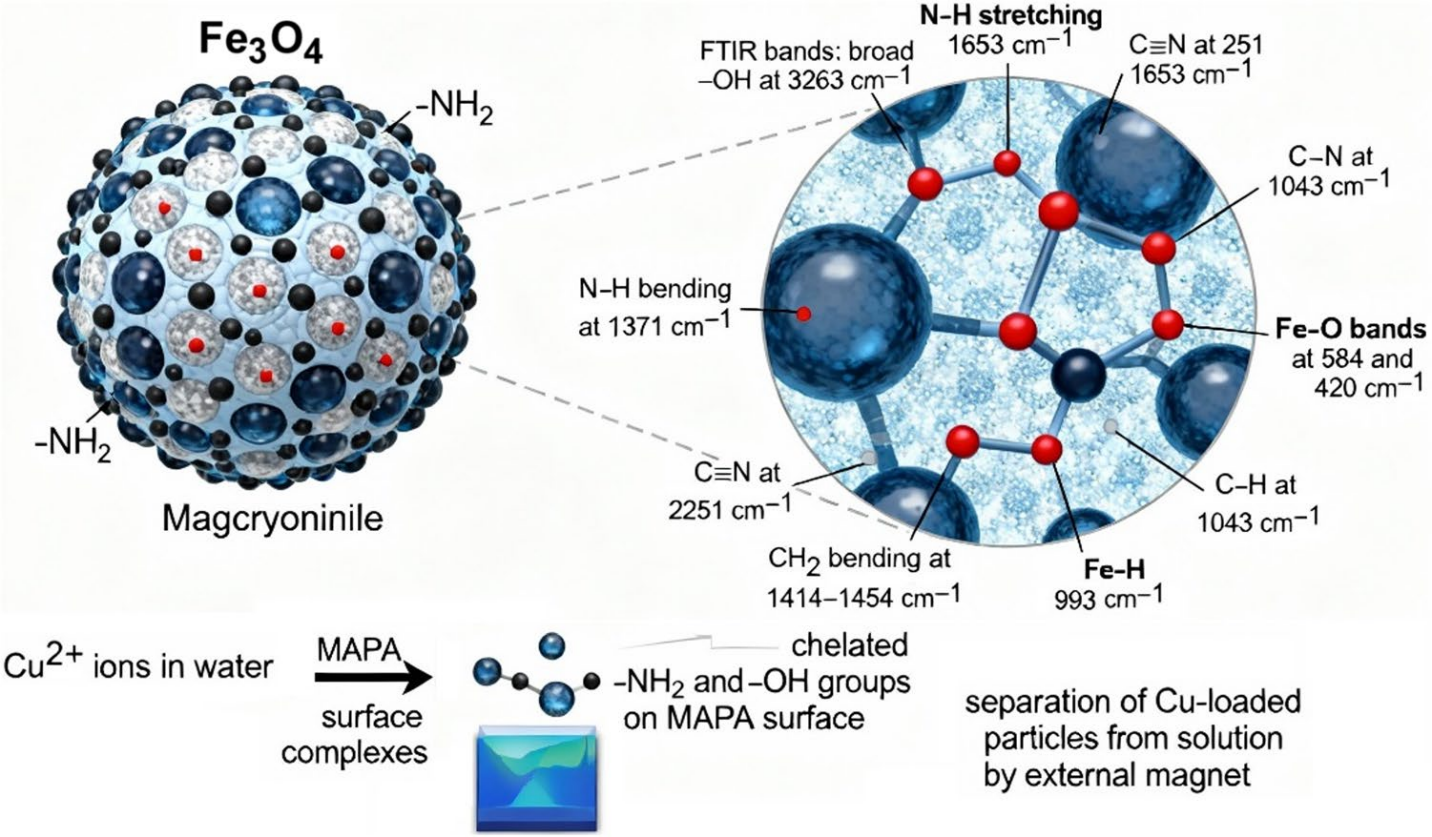
The most likely method for adsorbing the AO7 dye onto the prepared SNDB adsorbent.

The probable method by which MPAP absorbed the Cu^2+^ ions at 5.0 pH value is described in Fig. 18. The FTIR analysis shows the presence of various functional groups on the MPAP surface, including -NH_2_, -NH, -OH, -C ≡ N, -C-H, Fe-H and -CH_2_ groups. The XPS analyses proved the presence of Fe, Si and N doped in the prepared composite (MAPA) besides the presence of C and O. The adsorption mechanism of the dye ions in an acidic solution (pH 5.0) can be achieved by physical contact due to the electrostatic interaction between the Cu^2+^ ions and the nitrogen and oxygen lone pair on the MAPA surface^82,83^. The adsorption of ionizable organic molecules to the positively charged surface of the MPAP is the most significant process, and it is mediated by electrostatic interactions. How successfully an aqueous solution attracts or repels impurities depends on its pH and ionic strength^84–89^.

## Conclusion

Using batch experiments, a magnetic amino polyacrylonitrile nanocomposite (MAPA) was created and used as an adsorbent to remove Cu^2+^ ions from aqueous solutions. Cu^2+^ ion removal from aqueous solutions was assessed using a magnetic amino polyacrylonitrile (MAPA) nanocomposite. The material, exhibiting a magnetization value of 11.098 emu/g, was fully investigated utilizing XRD, FTIR, XPS, BET, TGA, SEM, and VSM studies. Its effectiveness as an adsorbent was validated by the results, which showed that it performed best at pH 5.5, reaching a removal efficiency of 32.4%. Under the most advantageous experimental conditions, an initial Cu^2+^ ion concentration of 50 mg/L and an adsorbent dosage of 5.0 g/L, the nanocomposite reached a removal effectiveness of 78.3%. The maximal adsorption capacity (*q*_m_) was determined to be 5.65 mg/g. Kinetic modeling demonstrated that the adsorption process conformed to the Pseudo-second-order model (PSO), while equilibrium data were best represented by the Langmuir isotherm model (LIM), suggesting monolayer adsorption on a homogeneous surface. Furthermore, the nanocomposite exhibited reusability, as adsorbed Cu^2+^ ions could be effectively desorbed using sodium hydroxide, highlighting its cost-effectiveness for practical water treatment applications. RSM was used to optimize the parameters, and the results showed that utilizing 5.12 g of the MAPA adsorbent and 51.51 mg/L of Cu^2+^ solution in 58.6 min could get the highest degradation percentage (75.65%). The MAPA nanocomposite demonstrates strong Cu^2+^ adsorption capacity, magnetic recoverability, and process optimization proven by ANN and RSM modeling. Future studies can test the feasibility of using this innovative material in expansion, its application to other metals, and the environmental sustainability of this material.

## Data Availability

The corresponding author of the research can provide the datasets used in this study for review upon request.

## References

[1] Jomova, K., Alomar, S. Y., Nepovimova, E., Kuca, K. & Valko, M. Heavy metals: toxicity and human health effects. *Arch. Toxicol.* **99** (1), 153–209 (2025). 39567405 10.1007/s00204-024-03903-2 PMC11742009

[2] Sharma, M., Kant, R., Sharma, A. K. & Sharma, A. K. Exploring the impact of heavy metals toxicity in the aquatic ecosystem. *Int. J. Energy Water Resour.* **9** (1), 267–280 (2025).

[3] Mitra, S. et al. Impact of heavy metals on the environment and human health: novel therapeutic insights to counter the toxicity. *J. King Saud University-Science*. **34** (3), 101865 (2022).

[4] Ohiagu, F. O., Chikezie, P. C., Ahaneku, C. C. & Chikezie, C. M. Human exposure to heavy metals: toxicity mechanisms and health implications. *Mater. Sci. Eng. Int. J.* **6** (2), 78–87 (2022).

[5] Abd Elnabi, M. K. et al. Toxicity of heavy metals and recent advances in their removal: a review. *Toxics* **11** (7), 580 (2023). 37505546 10.3390/toxics11070580 PMC10384455

[6] Hussain, A., Madan, S. & Madan, R. Removal of heavy metals from wastewater by adsorption. *Heavy Metals—Their Environ. Impacts Mitigation*. 10.5772/intechopen.95841 (2021).

[7] Qasem, N. A., Mohammed, R. H. & Lawal, D. U. Removal of heavy metal ions from wastewater: a comprehensive and critical review. *Npj Clean. Water*. **4** (1), 36 (2021).

[8] Shrestha, R. et al. Technological trends in heavy metals removal from industrial wastewater: A review. *J. Environ. Chem. Eng.* **9** (4), 105688 (2021).

[9] Khanzada, A. K. et al. Hydrochar as a bio-based adsorbent for heavy metals removal: A review of production processes, adsorption mechanisms, kinetic models, regeneration and reusability. *Sci. Total Environ.* **945**, 173972 (2024). 38897477 10.1016/j.scitotenv.2024.173972

[10] Maftouh, A., El Fatni, O., Hajjaji, E., Jawish, S. & Sillanpää, M. M.W. Comparative review of different adsorption techniques used in heavy metals removal in water. *Biointerface Res. Appl. Chem.* **13** (4) (2023).

[11] Rajendran, S. et al. A critical and recent developments on adsorption technique for removal of heavy metals from wastewater-A review. *Chemosphere* **303**, 135146 (2022). 35636612 10.1016/j.chemosphere.2022.135146

[12] Pan, B. et al. Development of polymeric and polymer-based hybrid adsorbents for pollutants removal from waters. *Chem. Eng. J.* **151** (1–3), 19–29 (2009).

[13] Ullah, R. et al. Sustainable clay-polymer adsorbents for emerging contaminants removal: a review. *Int. J. Environ. Anal. Chem.* 1–23 (2025).

[14] Arabsorkhi, B., Sereshti, H. & Abbasi, A. Electrospun metal–organic framework/polyacrylonitrile composite nanofibrous mat as a microsorbent for the extraction of Tetracycline residue in human blood plasma. *J. Sep. Sci.* **42** (8), 1500–1508 (2019). 30730108 10.1002/jssc.201801305

[15] Bansal, P. & Purwar, R. Polyacrylonitrile/clay nanofibrous nanocomposites for efficient adsorption of cr (VI) ions. *J. Polym. Res.* **28** (1), 7 (2021).

[16] Sun, D. W., Li, Y. F., Zhang, B. & Pan, X. B. Preparation and characterization of novel nanocomposites based on polyacrylonitrile/kaolinite. *Compos. Sci. Technol.* **70** (6), 981–988 (2010).

[17] Yang, X. et al. Ball-milled dysprosium oxide loaded biochar-montmorillonite composite for efficient removal and great recycling performance of cationic organic pollutants. *Industrial Crops Prod.* **235**, 121777. 10.1016/j.indcrop.2025.121777 (2025).

[18] Xue, Y. et al. Bio-based benzoxazine containing phthalonitrile: nonsolvent synthesis, curing behavior and pyrolysis mechanism. *Polym. Degrad. Stab.* **241**, 111542. 10.1016/j.polymdegradstab.2025.111542 (2025).

[19] Motaghi, H., Arabkhani, P., Parvinnia, M., Javadiand, H. & Asfaram, A. Synthesis of a highly porous three-dimensional PVA/GO/ZIF-67 cryogel for the simultaneous treatment of water contaminated with cadmium(II) and lead(II) heavy metal ions. *New. J. Chem.* **46**, 4449–4461. 10.1039/d1nj05418j (2022).

[20] Esfandian, H., Javadian, H., Parvini, M., Khoshandam, B. & Katal, R. Batch and column removal of copper by modified brown algae sargassum Bevanom from aqueous solution. *Asia-Pac J. Chem. Eng.* **8**, 665–678. 10.1002/apj.1707 (2013).

[21] Verma, M. et al. Capturing of inorganic and organic pollutants simultaneously from complex wastewater using recyclable magnetically Chitosan functionalized with EDTA adsorbent. *Process Saf. Environ. Prot.* **167**, 56–66. 10.1016/j.psep.2022.09.009 (2022).

[22] Verma, M. et al. One-step functionalization of Chitosan using EDTA: kinetics and isotherms modeling for multiple heavy metals adsorption and their mechanism. *J. Water Process. Eng.* **49**, 102989. 10.1016/j.jwpe.2022.102989 (2022).

[23] Verma, M. et al. Enhanced removal and Stepwise recovery of inorganic and organic micropollutants from water using novel graphene oxide doped multifunctional β-cyclodextrin Chitosan polymer. *J. Phys. Chem. Solids*. **182**, 111615. 10.1016/j.jpcs.2023.111615 (2023).

[24] Verma, M. et al. Simultaneous capturing of mixed contaminants from wastewater using novel one-pot Chitosan functionalized with EDTA and graphene oxide adsorbent. *Environ. Pollut.* **304**, 119130. 10.1016/j.envpol.2022.119130 (2022). 35331798 10.1016/j.envpol.2022.119130

[25] Verma, M., Lee, I., Hong, Y., Kumar, V. & Kim, H. Multifunctional β-Cyclodextrin-EDTA-Chitosan polymer adsorbent synthesis for simultaneous removal of heavy metals and organic dyes from wastewater. *Environ. Pollut.* **292**, 118447. 10.1016/j.envpol.2021.118447 (2022). 34742823 10.1016/j.envpol.2021.118447

[26] Zhang, K. et al. Magnetic high-swelling cyclodextrin polymer adsorbent for rapid removal of pollutants and efficient recovery from water. *Sep. Purif. Technol.* **366**, 132741 (2025).

[27] He, J., Zhu, L., Guo, S. & Yang, B. An effective strategy for coal-series Kaolin utilization: Preparation of magnetic adsorbent for congo red adsorption. *Chem. Eng. Sci.* **304**, 120958 (2025).

[28] Zhao, X. et al. Removal of fluoride from aqueous media by Fe_3_O_4_@Al(OH)_3_ magnetic nanoparticles. *J. Hazard. Mater.* **173** (1–3), 102–109 (2010). 19747775 10.1016/j.jhazmat.2009.08.054

[29] Aksu, Z., Egretli, G. & Kutsal, T. A comparative study of copper (II) biosorption on Ca-alginate, agarose and immobilized C. vulgaris in a backed-bed column. *Proc. Biochem.* **33** (4), 393–400 (1998).

[30] El-Sikaily, A., El Nemr, A. & Khaled, A. Copper sorption onto dried red Alga *Pterocladia capillacea* and its activated carbon. *Chem. Eng. J.* **168**, 707–714. 10.1016/j.cej.2011.01.064 (2011).

[31] Al-Bulushi, N. I., King, P. R., Blunt, M. J. & Kraaijveld, M. Artificial neural networks workflow and its application in the petroleum industry. *Neural Comput. Appl.* **21**, 409–421. 10.1007/s00521-010-0501-6 (2010).

[32] Ghaedi, A. M. & Vafaei, A. Applications of artificial neural networks for adsorption removal of dyes from aqueous solution: A review, adv. *Colloid Interface Sci.* **245**, 20–39. 10.1016/J.CIS.2017.04.015 (2017). 10.1016/j.cis.2017.04.015 28473053

[33] Takai, Z. I., Mustafa, M. K., Asman, S. & Sekak, K. A. Preparation and characterization of magnetite (Fe_3_O_4_) nanoparticles by sol-gel method. *Int. J. Nanoelectron Mater.* **12** (1), 37–46 (2019).

[34] Leslie-Pelecky, D. L. & Rieke, R. D. Magnetic properties of nanostructured materials. *Chem. Mater.* **8** (8), 1770–1783 (1996).

[35] Dastjerdi, O. D., Shokrollahi, H. & Mirshekari, S. A review of synthesis, characterization, and magnetic properties of soft spinel ferrites. *Inorg. Chem. Commun.* **153**, 110797 (2023).

[36] Li, W. et al. Microstructure and magnetic properties of the fesial soft magnetic composite with a NiFe_2_O_4_-doped phosphate insulation coating. *J. Alloys Compd.* **960**, 171010 (2023).

[37] Elamin, N. Y., Modwi, A., Abd El-Fattah, W. & Rajeh, A. Synthesis and structural of Fe_3_O_4_ magnetic nanoparticles and its effect on the structural optical, and magnetic properties of novel Poly (methyl methacrylate)/Polyaniline composite for electromagnetic and optical applications. *Opt. Mater.* **135**, 113323 (2023).

[38] Ryu, J. et al. Room-temperature crosslinkable natural polymer binder for high‐rate and stable silicon anodes. *Adv. Funct. Mater.* **30** (9), 1908433 (2020).

[39] Zhang, S., Li, X. Y. & Chen, J. P. An XPS study for mechanisms of arsenate adsorption onto a magnetite-doped activated carbon fiber. *J. Colloid Interface Sci.* **343** (1), 232–238 (2010). 20018292 10.1016/j.jcis.2009.11.001

[40] Toupin, M. & Bélanger, D. Spontaneous functionalization of carbon black by reaction with 4-nitrophenyldiazonium cations. *Langmuir* **24** (5), 1910–1917 (2008). 18211105 10.1021/la702556n

[41] Nosratabad, N. A., Yan, Q., Cai, Z. & Wan, C. Exploring nanomaterial-modified Biochar for environmental remediation applications. *Heliyon* **10**, e37123 (2024). 10.1016/j.heliyon.2024.e37123 PMC11417198 39315228

[42] Broujenia, B. R. & Nilchib, A. Preparation and characterization of polyacrylonitrile nanofiber adsorbent modified with 6-amino-1-hexanethiol hydrochloride for the adsorption of thorium (IV) ion from aqueous solution. *Desalination Water Treat.* **133**, 122–133 (2018).

[43] Toncón Leal, C. F., Villarroel Rocha, J., Silva, M. T. P., Braga, T. P. & Sapag, K. Characterization of mesoporous region by the scanning of the hysteresis loop in adsorption–desorption isotherms. *Adsorption* **27**, 1109–1122 (2021).

[44] Inglezakisa, V. J., Poulopoulosa, S. G. & Kazemian, H. Insights into the S-shaped sorption isotherms and their dimensionless forms. *Microporous Mesoporous Mater.* **272**, 166–176 (2018).

[45] Sing, K. S. Reporting physisorption data for gas/solid systems with special reference to the determination of surface area and porosity (Recommendations 1984). *Pure Appl. Chem.* **57** (4), 603–619 (1985).

[46] Zarnegar, Z. & Safari, J. Modified chemical coprecipitation of magnetic magnetite nanoparticles usinglinear–dendritic copolymers. *Green Chem. Lett. Rev.* **10** (4), 235–240 (2017).

[47] Parajuli, K., Sah, A. K. & Paudyal, H. Green synthesis of magnetite nanoparticles using aqueous leaves extracts of Azadirachta indica and its application for the removal of As(V) from water. *Green. Sustainable Chem.* **10**, 117–132 (2020).

[48] El-Alaily, T. M. et al. Construction and calibration of a low cost and fully automated vibrating sample magnetometer. *J. Magn. Magn. Mater.* **386**, 25–30 (2015).

[49] Nisticò, R., Cesano, F. & Garello, F. Magnetic materials and systems: domain structure visualization and other characterization techniques for the application in the materials science and biomedicine. *Inorganics* **8**, 6 (2020).

[50] Neisan, R. S., Saady, N. M. C., Bazan, C., Zendehboudi, S. & Albayati, T. M. Adsorption of copper from water using TiO_2_-modified activated carbon derived from orange peels and date seeds: response surface methodology optimization. *Heliyon* **9**, e21420 (2023). 38027893 10.1016/j.heliyon.2023.e21420 PMC10660060

[51] Luo, H., Liu, Y., Lu, H., Fang, Q. & Rong, H. Efficient adsorption of Tetracycline from aqueous solutions by modified alginate beads after the removal of Cu (II) ions. *ACS Omega*. **6**, 6240–6251 (2021). 33718714 10.1021/acsomega.0c05807 PMC7948232

[52] Shoaib, A. G. M., Ragab, S., El Sikaily, A., Yılmaz, M. & Nemr, E. Thermodynamic, kinetic, and isotherm studies of direct blue 86 dye absorption by cellulose hydrogel. *Sci. Rep.* **4**, 11 (2023). 10.1038/s41598-023-33078-2 PMC10090095 37041227

[53] Hassaan, M. A. et al. Improved methylene blue adsorption from an aqueous medium by ozone-triethylenetetramine modification of sawdust-based Biochar. *Sci. Rep.* **8**, 1 (2023). 10.1038/s41598-023-39495-7 PMC10394039 37528164

[54] Kargi, F. & Cikla, S. Biosorption of zinc(II) ions onto powdered waste sludge (PWS): kinetics and isotherms. *Enzyme Microb. Technol.* **38**, 705–710. 10.1016/j.enzmictec.2005.11.005 (2006).

[55] Hamdaoui, O. Batch study of liquid-phase adsorption of methylene blue using Cedar sawdust and crushed brick. *J. Hazard. Mater.* **135**, 264–273. 10.1016/j.jhazmat.2005.11.062 (2006). 16406337 10.1016/j.jhazmat.2005.11.062

[56] Annadurai, G., Juang, R. S. & Lee, D. J. Use of cellulose-based wastes for adsorption of dyes from aqueous solutions. *J. Hazard. Mater. 92*. **3**, 263–274. 10.1016/S0304-3894(02)00017-1 (2002). 10.1016/s0304-3894(02)00017-1 12031611

[57] Weber, W. J. & Morris, J. C. Kinetics of adsorption on carbon from solution. *J. Sanit. Eng. Div.* **89**, 31–60 (1963).

[58] Boyd, G. E., Adamson, A. W. & Myers, S. Jr. The exchange adsorption of ions from aqueous solutions by organic zeolites. *II Kinetics J. Am. Chem. Soc.* **69**, 2836–2848. 10.1021/ja01203a066 (1947). 10.1021/ja01203a066 20270838

[59] El-Nemr, M. A. et al. Fabrication of pea pods biochar-NH_2_ (PBN) for the adsorption of toxic Cr^6+^ ion from aqueous solution. *Appl. Water Sci.* **13**, 194 (2023).

[60] Elkatory, M. R., Yılmaz, M., Hassaan, M. A. & El Nemr, A. Fabrication of date palm kernel biochar-sulfur (DPKB-S) for super adsorption of methylene blue dye from water. *Sci. Rep.* **3**, 21 (2024). 10.1038/s41598-024-56939-w PMC10958023 38514691

[61] Yu, B., Shukla, Y. Z. A., Shukla, S. S. & Dorris, K. L. The removal of heavy metals from aqueous solutions by sawdust adsorption— removal of lead and comparison of its adsorption with copper. *J. Hazard. Mater. B*. **84**, 83–94 (2001). 10.1016/s0304-3894(01)00198-4 11376886

[62] Huang, C. P., Huang, C. P. & Morehart, A. L. The removal of Cu(II) from dilute aqueous solutions by Saccharomyces cerevisiae. *Water Res.* **24**, 433–439 (1990).

[63] Annadurai, G., Juang, R. S. & Lee, D. J. Adsorption of heavy metals from water using banana and orange peels. *Water Sci. Technol.* **47** (1), 185–190 (2003). 12578193

[64] Razmovski, R. & Sciban, M. Biosorption of Cr(VI) and Cu(II) by waste tea fungal biomass. *Ecol. Eng.* **34**, 179–186 (2008).

[65] Lujan, J. R., Darnall, D. W., Stark, P. C., Rayson, G. D. & Gardea-Torresdey, J. L. Metal ion binding by algae and higher plant tissues. *Solvent Extr. Ion Exch.* **12** (4) (1994).

[66] Liu, C., Ngo, H. H., Guo, W. & Tung, K. L. Optimal conditions for Preparation of banana peels, sugarcane Bagasse and watermelon rind in removing copper from water. *Bioresour. Technol.* **119**, 349–354 (2012). 22750502 10.1016/j.biortech.2012.06.004

[67] Ho, Y. S. Removal of copper ions from aqueous solution by tree fern. *Water Res.* **37**, 2323–2330 (2003). 12727241 10.1016/S0043-1354(03)00002-2

[68] Khan, J. et al. Removal of copper ions from wastewater via adsorption on modified hematite (α-Fe_2_O_3_) iron oxide coated sand. *J. Clean. Prod.* **319**, 128687. 10.1016/j.jclepro.2021.128687 (2021).

[69] Zafar, S. et al. Removal of copper ions from aqueous solution using NaOH-treated rice husk. *Emergent Mater.* **3**, 857–870. 10.1007/s42247-020-00126-w (2020).

[70] Sulaiman, S. et al. Adsorptive removal of copper (II) ions from aqueous solution using a magnetite Nano–Adsorbent from mill scale waste: Synthesis, Characterization, adsorption and kinetic modelling studies. *Nanoscale Res. Lett.* **16**, 168. 10.1186/s11671-021-03622-y (2021). 34837537 10.1186/s11671-021-03622-y PMC8627547

[71] Duyen, L. T. & Bac, B. H. Adsorption–desorption behavior of Halloysite clay for Cu^2+^ ions and recovery of copper by electrodeposition method. *Desalination Water Treat.* **317**, 100207. 10.1016/j.dwt.2024.100207 (2024).

[72] Bishop, C. M. *Neural Networks for Pattern Recognition* (Oxford University Press, 1995).

[73] Jiang, G., Keller, J., Bond, P. L. & Yuan, Z. Predicting concrete corrosion of sewers using artificial neural network. *Water Res.* **92**, 52–60 (2016). 26841228 10.1016/j.watres.2016.01.029

[74] Riyanti, F., Hasanudin, H., Rachmat, A., Purwaningrum, W. & Hariani, P. L. Photocatalytic degradation of methylene blue and congo red dyes from aqueous solutions by bentonite-Fe_3_O_4_ magnetic. *Commun. Sci. Technol.* **8** (1), 1–9. 10.21924/cst.8.1.2023.1007 (2023).

[75] Meky, A. I., Hassaan, M. A., Fetouh, H. A., Ismail, A. M. & Nemr, E. A. Hydrothermal fabrication, characterization and RSM optimization of cobalt-doped zinc oxide nanoparticles for antibiotic photodegradation under visible light. *Sci. Rep.* **14** (1), (2016). 10.1038/s41598-024-52430-8 (2024). 10.1038/s41598-024-52430-8 PMC11231344 38263230

[76] Hassaan, M. A., Meky, A. I., Fetouh, H. A., Ismail, A. M. & Nemr, E. Central composite design and mechanism of antibiotic Ciprofloxacin photodegradation under visible light by green hydrothermal synthesized cobalt-doped zinc oxide nanoparticles. *Sci. Rep.* **14** (1), 9144. 10.1038/s41598-024-58961-4 (2024). 38644378 10.1038/s41598-024-58961-4 PMC11551219

[77] Hassaan, M. A. et al. Synthesis, characterization, optimization and application of Pisum sativum peels S and N-doping biochars in the production of biogas from Ulva lactuca. *Renew. Energy*. **221**, 119747. 10.1016/j.renene.2023.119747 (2024).

[78] Hassaan, M. A. et al. Box-Behnken design and life cycle assessment for nickel oxide nanoparticles application in biomethane production. *Chem. Eng. J.* **474**, 145924. 10.1016/j.cej.2023.145924 (2023).

[79] Hassaan, M. A. et al. Application of multi-heteroatom doping Biochar in a newly proposed mechanism of electron transfer in biogas production. *Chem. Eng. J.* **470**, 144229. 10.1016/j.cej.2023.144229 (2023).

[80] Meky, A. I., Hassaan, M. A., Fetouh, H. A., Ismail, A. M. & Nemr, E. Cube-shaped Cobalt-doped zinc oxide nanoparticles with increased visible-light-driven photocatalytic activity achieved by green co-precipitation synthesis. *Sci. Rep.* **13** (1), 19329. 10.1038/s41598-023-46464-7 (2023). 37935868 10.1038/s41598-023-46464-7 PMC10630306

[81] Ragab, S., Elkatory, M. R., Hassaan, M. A. & El Nemr, A. Experimental, predictive and RSM studies of H_2_ production using Ag-La-CaTiO_3_ for water-splitting under visible light. *Sci. Rep.* **14** (1), 1019. 10.1038/s41598-024-51219-z (2024). 38200036 10.1038/s41598-024-51219-z PMC10781765

[82] El-Nemr, M. A., Abdelmonem, N. M., Ismail, I. M., Ragab, S. & El Nemr, A. Ozone and ammonium hydroxide modification of Biochar prepared from Pisum sativum peels improves the adsorption of copper (II) from an aqueous medium. *Environ. Process.* **7**, 973–1007 (2020a).

[83] El-Nemr, M. A., Abdelmonem, N. M., Ismail, I. M., Ragab, S. & El Nemr, A. The efficient removal of the hazardous Azo dye acid orange 7 from water using modified Biochar from pea peels. *Desalin. Water Treat.* **203**, 327–355 (2020b).

[84] Eleryan, A. et al. Copper (II) ion removal by chemically and physically modified sawdust Biochar. *Biomass Convers. Bioref*. **14**, 9283–9320 (2024a).

[85] Eleryan, A. et al. Kinetic and isotherm studies of acid orange 7 dye absorption using sulphonated Mandarin Biochar treated with TETA. *Biomass Convers. Biorefin*. **14** (9), 10599–10610 (2024b).

[86] Eleryan, A. et al. Mandarin biochar-CO-TETA was utilized for acid red 73 dye adsorption from water, and its isotherm and kinetic studies were investigated. *Sci. Rep.* **14** (1), 13021 (2024c). 38844483 10.1038/s41598-024-62870-x PMC11156941

[87] Eleryan, A. et al. Isothermal and kinetic screening of Methyl red and Methyl orange dyes adsorption from water by Delonix regia biochar-sulphur oxide (DRB-SO). *Sci. Rep.* **14** (1), 13585 (2024d). 38866857 10.1038/s41598-024-63510-0 PMC11169550

[88] Eleryan, A. et al. Isothermal and kinetic screening of Methyl red and Methyl orange dyes adsorption from water by Delonix regia biochar-sulphur oxide (DRB-SO). *Sci. Rep.* **14**, 13585 (2024e). 38866857 10.1038/s41598-024-63510-0 PMC11169550

[89] Eleryan, A. et al. Mandarin Biochar-TETA (MBT) prepared from *Citrus reticulata* peels for adsorption of acid yellow 11 dye from water. *Sci. Rep.* **12**, 17797 (2022). 36273033 10.1038/s41598-022-22359-x PMC9587999

